# CD38-Induced Metabolic Dysfunction Primes Multiple Myeloma Cells for NAD^+^-Lowering Agents

**DOI:** 10.3390/antiox12020494

**Published:** 2023-02-15

**Authors:** Pamela Becherini, Debora Soncini, Silvia Ravera, Elisa Gelli, Claudia Martinuzzi, Giulia Giorgetti, Antonia Cagnetta, Fabio Guolo, Federico Ivaldi, Maurizio Miglino, Sara Aquino, Katia Todoerti, Antonino Neri, Andrea Benzi, Mario Passalacqua, Alessio Nencioni, Ida Perrotta, Maria Eugenia Gallo Cantafio, Nicola Amodio, Antonio De Flora, Santina Bruzzone, Roberto M. Lemoli, Michele Cea

**Affiliations:** 1 Clinic of Hematology, Department of Internal Medicine and Medical Specialties (DiMI), University of Genoa, 16126 Genoa, Italy; 2 Department of Experimental Medicine (DIMES), University of Genoa, 16126 Genoa, Italy; 3 IRCCS Ospedale Policlinico San Martino, 16132 Genoa, Italy; 4 Department of Internal Medicine (DiMI), University of Genoa, 16126 Genoa, Italy; 5 Hematology and Hematopoietic Stem Cell Transplantation Unit, IRCCS Ospedale Policlinico San Martino, 16132 Genoa, Italy; 6 Pathology and Laboratory Medicine, Fondazione IRCCS Istituto Nazionale dei Tumori di Milan, 20133 Milano, Italy; 7 Scientific Directorate, Azienda USL-IRCCS di Reggio Emilia, 42122 Reggio Emilia, Italy; 8 Department of Biology, Ecology and Earth Sciences, Centre for Microscopy and Microanalysis (CM2), University of Calabria, Arcavacata di Rende, 87036 Cosenza, Italy; 9 Department of Experimental and Clinical Medicine, Magna Graecia University, 88100 Catanzaro, Italy

**Keywords:** NAD^+^ biosynthetic pathway, NAD^+^-lowering agents, cancer metabolism, mitochondrial disfunction, oxidative stress, multiple myeloma

## Abstract

Cancer cells fuel growth and energy demands by increasing their NAD^+^ biosynthesis dependency, which therefore represents an exploitable vulnerability for anti-cancer strategies. CD38 is a NAD^+^-degrading enzyme that has become crucial for anti-MM therapies since anti-CD38 monoclonal antibodies represent the backbone for treatment of newly diagnosed and relapsed multiple myeloma patients. Nevertheless, further steps are needed to enable a full exploitation of these strategies, including deeper insights of the mechanisms by which CD38 promotes tumorigenesis and its metabolic additions that could be selectively targeted by therapeutic strategies. Here, we present evidence that CD38 upregulation produces a pervasive intracellular-NAD^+^ depletion, which impairs mitochondrial fitness and enhances oxidative stress; as result, genetic or pharmacologic approaches that aim to modify CD38 surface-level prime MM cells to NAD^+^-lowering agents. The molecular mechanism underlying this event is an alteration in mitochondrial dynamics, which decreases mitochondria efficiency and triggers energetic remodeling. Overall, we found that CD38 handling represents an innovative strategy to improve the outcomes of NAD^+^-lowering agents and provides the rationale for testing these very promising agents in clinical studies involving MM patients.

## 1. Introduction

Multiple myeloma (MM) is a blood tumor characterized by the clonal proliferation of malignant plasma cells in the bone marrow microenvironment, monoclonal protein in the blood or urine, and associated organ dysfunction [1,2]. The past decade has seen remarkable progress in the management of MM patients thanks to the introduction of novel therapies, including proteasome inhibitors (PIs), immunomodulatory agents (iMIDs), and monoclonal antibodies (MoAbs). However, despite the unquestionable clinical benefits, MM still remains an incurable disease [3]. Therefore, there is an unmet medical need to both enhance the efficacy of existing treatments and to identify novel therapeutic targets as well [4]. MM is a highly heterogeneous disease, which shares many features with other tumors, including the ability to readjust intracellular metabolism to sustain their needs for growth and proliferation [5]. As result, energetic remodeling represents a hallmark which could also be therapeutically exploited in this setting. Nicotinamide adenine dinucleotide (NAD^+^) is the main co-factor associated with several cellular metabolic pathways, such as glycolysis, tricarboxylic acid cycle (TCA) cycle, and oxidative phosphorylation (OxPhos) [6]. Additionally, it serves as a substrate for numerous NAD^+^-degrading enzymes, including poly (ADP-ribose) polymerases (PARPs), mono (ADP-ribose) transferases (ARTs), NAD^+^ase/ADP-ribosyl cyclases (CD38/CD157), and sirtuins (SIRT). Remarkably, through these enzymes, NAD^+^ is involved in a wide-range of cellular biological processes: proliferation, adhesion, calcium mobilization, DNA damage, cell cycle control, and transcriptional regulation [7]. As result, the inhibition of NAD^+^ synthesis is detrimental for cancer cell survival and its targeting has received significant interest as anti-tumor strategy [8,9,10]. We previously reported that targeting nicotinamide phosphoribosyltransferase (NAMPT), the rate-limiting enzyme that catalyzes NAD^+^ synthesis from nicotinamide (NAM), is a promising approach to eliminate blood tumor cells that are highly dependent on NAD^+^, such as B-chronic lymphocytic leukemia (B-CLL), lymphomas, MM, and acute myeloid leukemia (AML) [11,12,13,14]. The current standard-of-care (SOC) for newly diagnosed (NDMM) and relapsed (MMRR) MM patients includes monoclonal antibodies targeting the NAD^+^-degrading enzyme CD38, thus supporting dependence on a sustained NAD^+^ biosynthesis also for this tumor [15,16,17,18,19,20]. Indeed, data derived from several clinical studies indicate CD38 as an attractive actionable target for MM, with the expectation that, by simultaneously interfering with several aspects of carcinogenesis, CD38-targeted agents have led to impressive clinical results. Nevertheless, further steps need to be taken to allow a full exploitation of CD38 as a target in MM, including a more thorough definition of the mechanisms by which it promotes tumorigenesis.

Here, we have investigated the metabolic events induced by CD38 upregulation in MM cells by focusing on their effects on the anti-tumor activity of NAD^+^-lowering agents. In detail, we show that the pervasive energetic depletion triggered by CD38 upregulation results in mitochondrial fitness impairment and oxidative stress enhancement. Remarkably, this metabolic reprogramming, which occurs regardless of specific CD38-handling strategy (genetic or drug-induced), makes MM cells more vulnerable to NAD^+^-depleting agents with massive levels of cell death. Overall, these proof-of-principle experiments demonstrate the critical role of mitochondrial dynamic dysfunction in CD38-upregulated activity and support use of this approach to prime MM cells to NAD^+^-lowering agents.

## 2. Materials and Methods

Detailed protocols for all sections are described in the Supplementary Materials.

### 2.1. Reagents

FK866 and GMX1778 were purchased from Sigma Aldrich while All-trans Retinoic Acid (ATRA), Lenalidomide (LEN), and Pomalidomide (POM) were purchased from Selleck Chemicals. Drugs were dissolved in dimethylsulfoxide (DMSO) and subsequently diluted in cell culture medium (10% fetal bovine serum) immediately before use. The maximum final concentration of DMSO (<0.1%) did not affect cell proliferation and did not induce cytotoxicity on the cell lines tested.

### 2.2. Cell Lines and Culture

The HMCLs were purchased from ATCC or DSMZ. All cell lines were mycoplasma-free and were routinely tested for it. Cells were cultured in RPMI-1640 medium containing 10% fetal bovine serum (FBS; GIBCO), 4 mM glutamine, 100 U mL^−1^ penicillin, and 100 μg mL^−1^ streptomycin (GIBCO). The 293T cells were cultured in DMEM high glucose containing 10% FBS (GIBCO), 4 mM glutamine, 100 U mL^−1^ penicillin, and 100 μg mL^−1^ streptomycin (GIBCO).

### 2.3. Primary Cells

Peripheral blood samples collected from healthy volunteers were processed through Ficoll-Paque (GE Healthcare) gradient to obtain PBMCs. Malignant cells from individuals affected by MM were purified from bone marrow samples after informed consent was obtained in accordance with the Declaration of Helsinki and approval by the Ethical Committee at IRCCS AOU San Martino-IST, Genoa, Italy (Prot. IRSTB100/INTHEMA). Mononuclear cells were separated using Ficoll-Paque density sedimentation, and plasma cells were purified by positive selection (>95% CD138+) with an anti–CD138 magnetic activated cell separation microbeads system (Miltenyi).

### 2.4. Lentiviral Mediated Gene Transfer

pLV[Exp]-CMV > EGFP: T2A: Puro (pLV empty vector) and pLV[Exp]-EGFP: T2A: Puro-SV40 > hCD38 (CD38-overexpressing plasmid) were used to create stable isogenic MM cell lines. All lentiviral plasmids were purchased from Vector Builder.

### 2.5. Measurement of CD38 Enzymatic Activity

Cyclase activity was evaluated by measuring the production of cyclic GDP-ribose from NGD^+^, the NAD^+^ analogue, as previously described [21].

### 2.6. Determination of Intracellular NAD^+^

The NAD^+^ content of whole cells, isolated mitochondria, was assessed as described in [22].

### 2.7. Mitochondria Fractionation

Cells were homogenized in a 10 mmol/l Tris/HCl buffer, pH 7.8, containing 0.32 M sucrose and 1 mmol/1 EDTA using a Dounce glass homogenizer (Wheaton, Millville, NJ, USA). After the separation of nuclei, mitochondria were collected by centrifugation at 13,000× *g* and 4 °C for 20 min. The mitochondrial pellet was re-suspended in a trehalose buffer [23] at a final concentration of 100 μg/mL.

### 2.8. Western Blotting

Whole-cell lysates and SDS-page electrophoretic separation and blotting were performed as previously described [24]. Immunoblotting was assessed using the following primary antibodies: CD38 (Cell Signaling Technology Cat# 51000), NAMPT (Bethyl Cat#A300-372A), GAPDH (Cell Signaling Technology #5174S), SDHB (Abcam ab84622); anti-rabbit IgG HRP-linked antibody (Cell Signaling Technology Cat# 7074). Signals were obtained by a I bright™ 1500 Imaging System using standard ECL (Thermo Fisher, Waltham, MA, USA).

### 2.9. Statistical Analyses

All experiments were repeated at least three times and performed in triplicate; a representative experiment is shown in each figure. All data are shown as mean ± standard deviation (SD). Student’s *t*-test was used to compare two experimental groups using Graph-Pad Prism software 8.4.3 (http://www.graphpad.com). The minimal level of significance was specified as *p* < 0.05.

## 3. Results

### 3.1. CD38 Enzymatic Activity Affects NAD^+^ Intracellular Level and Influences Anti-MM Activity of NAD^+^-Depleting Agents

**Figure 1:**
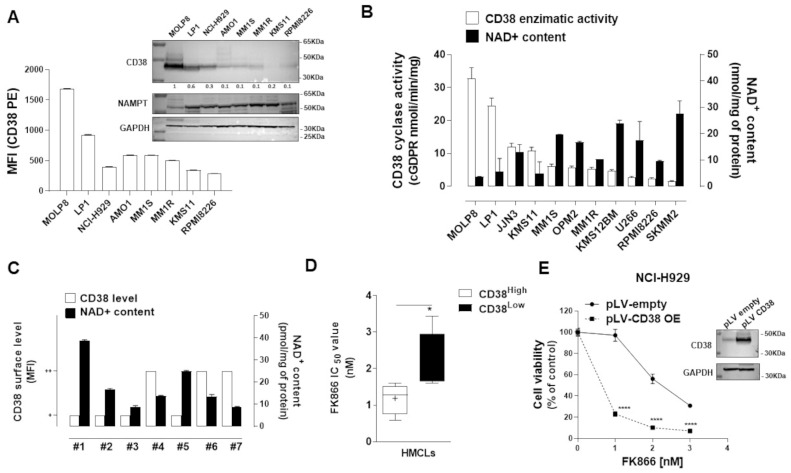
**CD38 enzymatic activity affects NAD^+^ intracellular level and influences anti-MM activity of NAD^+^-depleting agents.** (**A**) A panel of MM cell lines (HMCLs) were analyzed for CD38 and NAMPT protein levels by WB (**top panel**) or quantitative flow cytometry (**bottom panel**). One representative experiment is shown. (**B**) NAD^+^ content and CD38 enzymatic activity (GDP-ribosyl cyclase) were measured in indicated HMCLs. (**C**) NAD^+^ content and CD38 surface level (based on arbitrary units, as detailed in the Supplementary Materials) were measured in CD138+ cells derived from MM patients. (**D**) Relative expression of CD38 surface protein plotted versus FK866 cytotoxicity IC_50_ values: box plot showing cumulative results of MTS assays. (**E**) H929 cell line was lentivirally transduced with empty pLV and CD38-overexpressing pLV (CD38 OE) and then treated with increasing doses of FK866 (0–10 nM) for 96 h. Cell viability was measured with an MTS assay and presented as a percentage of control. Data are presented as mean ± S.D (*n* = 3) (* *p* ≤ 0.05, **** *p* ≤ 0.0001; unpaired *t* test).

Results derived from clinical trials have led anti-CD38 MoAbs-based regimens to become the SOC for MM patients’ treatment, both at diagnosis and relapse [15,17,19,25,26,27]. Nevertheless, further steps need to be taken for the full exploitation of CD38-targeting in MM, including a more comprehensive functional analysis of its impact on cell biology. Here, we initially screened CD38 cell-surface protein expression in a panel of MM cell lines (*n* = 8). Western blot and flow cytometry analyses both showed a heterogeneous trend of CD38 expression level, with MOLP8 and LP1 being the top-scored cell lines among those tested (Figure 1A). CD38 breaks down extracellular NAD^+^ to generate cyclic ADP-ribose (cADPR), ADP-ribose, and nicotinamide (Nam) [28]. As a result, we next screened the ability of MM cells to produce cGDPR from the NAD^+^ analog NGD^+^ and for their intracellular NAD^+^ content as well. As expected, consistent with the expression of a functional NAD^+^-metabolizing enzyme, cells with higher CD38 activity produced greater levels of cGDPR and carried lower NAD^+^ content (Figure 1B) with a significant inverse correlation (Figure S1). Purified CD138^+^ cells collected from MM patients (*n* = 7) also showed a similar trend regarding these parameters (Figure 1C).

NAD^+^ regulates many cancer-associated biological processes; therefore, the inhibition of its synthesis has been shown to be detrimental to wide range of cancer cells [11,12,13,14]. In such a scenario, we probed the impact of CD38 levels on the anti-MM activity of the chemical inhibitor of rate-limiting enzyme catalyzing NAD^+^ synthesis from nicotinamide (NAMPT), FK866. Greater sensitivity was observed among MM cell lines carrying higher CD38 levels, thus allowing us to hypothesize that the anti-MM activity of FK866 depends on CD38 levels (Figure 1D). As a result, we tested drug-sensitivity after CD38 handling: as expected, the higher activity of FK866 was found in CD38-overexpressing MM cells than the control (Figure 1E and Figure S2); similarly, another NAD^+^-lowering agent, GMX-1777, summarized these data (Figure S3). No significant differences were measured in the CD38-depleted cells (data not shown). Overall, our results show that CD38 level affects the anti-MM activity of NAD^+^-depleting agents and that hijacking CD38-NAD^+^ase activity would represent an innovative strategy to sensitize MM cells to these drugs.

### 3.2. Synergistic Effects of NAMPT Inhibitor Combined with CD38 Inducing Agents

**Figure 2:**
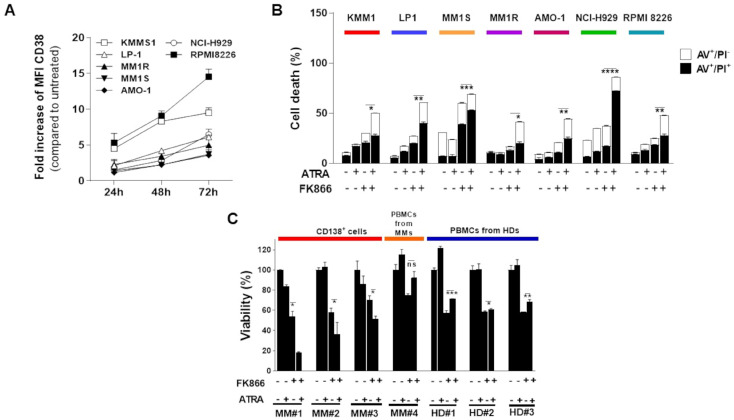
**Anti-MM synergistic effects of the NAMPT inhibitor FK866 combined with CD38 inducers.** (**A**) Indicated HMCLs were incubated with 10 nM ATRA for 24, 48, or 72 h and then analyzed by flow cytometry. The panel shows the fold increase in CD38 median fluorescence intensity (MFI) compared with control. (**B**) Apoptotic cell death assessed with flow cytometry analysis after Annexin V/PI staining in a panel of HMCLs treated with FK866 (3 nM), ATRA (3 nM) or their combination for 96 h. (**C**) CD138^+^ cells collected from three MM patients or PBMCs derived from one MM patient and three healthy donors (HDs) were treated with indicated doses of FK866 (3 nM), ATRA (1 nM) and their combination for 72 h. Cell viability was measured by MTS assay. Data in B and C are presented as mean ± S.D (*n* = 3). (ns *p* > 0.05, * *p* ≤ 0.05, ** *p* ≤ 0.01, *** *p* ≤ 0.001, **** *p* ≤ 0.0001; unpaired *t* test).

Given the clinical benefits of CD38-based anti-MM therapies, efforts have been made to upregulate its expression on plasma cells surface as a strategy to make results even more compelling [29,30,31,32,33,34,35]. Among these approaches, all-trans retinoic acid (ATRA) has shown remarkable efficacy in both preclinical and clinical settings [29]. Thus, after confirming ATRA ability to enhance CD38 cell-surface levels (Figure 2A and Figure S4), we tested the anti-MM activity of NAD^+^-lowering agents alone or in combination with this agent. As shown in Figure 2B and Figure S5A,B, the combination of low doses of ATRA and FK866 was synergistic in several MM cell lines, including those carrying a high risk cytogenetic features (LP1, MM1S, MM1R, and NCI-H929). Similar results were obtained with other CD38 modulators, such as lenalidomide (LEN) and pomalidomide (POM) [29,33], which are both IMiDs, by inducing a time- and dose-dependent CD38 upregulation (Figure S6A,B) and resulted in being synergic when combined with FK866 (Figure S6C–E). We next examined the effects of this drugs combination on primary plasma cells (PCs) collected from MM patients. As shown in Figure 2C, the enhanced anti-MM activity of this strategy was also preserved in these cells, regardless of specific disease stage, cytogenetic abnormalities, or previous drug-exposure (Table 1). Furthermore, although FK866 was also found to be slightly active on PBMCs derived from healthy donors or MM patients, probably due to the presence of activated T cells in these contexts [22], the combination did not work any better, which was consistent with its tumor specificity. Finally, to strengthen relevance of these findings, we tested also combinations on MM cells co-incubated with cytokines mixtures (IL-6 and IGF-1) mimicking bone-marrow milieu: anti-tumor activity was kept also in this context (Figure S7A,B). Overall, these data support CD38-handling as a pharmacological exploitable strategy for improving the anti-MM activity of NAD^+^-lowering agents.

### 3.3. FK866-Induced Transcriptomic Change among CD38-Overexpressing MM Patients Confers Better Prognosis

**Figure 3:**
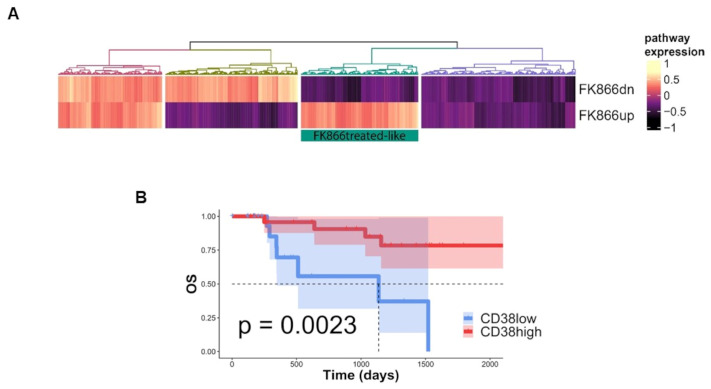
**FK866 transcriptomic changes among CD38-overexpressing MM patients confers survival advantage.** (**A**) Heatmap showing FK866 activity signature expression in NDMM patients derived from the CoMMpass dataset: a group of patients with gene expression in accordance with FK866 treatment is highlighted in the green rectangle (FK866 treated-like). (**B**) Kaplan–Meyer curves of the overall survival probability for FK866 treated-like patients were divided in quartiles for their expression of CD38. (FK866dn, signature created by FK866 down-regulated genes; FK866up, signature created by FK866 upregulated genes; CD38low, CD38 expression bottom quartile; CD38high, CD38 expression top quartile).

To support the translational relevance of in vitro data, an in-silico analysis was next employed. The FK866-induced transcriptome changes derived from GSE96636 [36] were validated among MM patients by using a large cohort of NDMM included in the MMRF CoMMpass dataset. An enrichment analysis identified a group of MM patients with an FK866-specific signature (FK866up and FK866dn, respectively) resembling drug-treated cells (FK866 treated-like) (Figure 3A and Table 2) among those with a higher expression of CD38 (top quartiles) that had a prognostic advantage over those with lower levels, in terms of overall survival (Figure 3B; *p* = 0.0023). Overall, these data support clinical benefits deriving from the NAD^+^-lowering agent’s exposure to MM patients carrying higher levels of CD38.

### 3.4. NAD^+^ Depletion Accounts for the Enhanced Sensibility of CD38-Upregulated MM Cells to Nampt-Inhibitors

**Figure 4:**
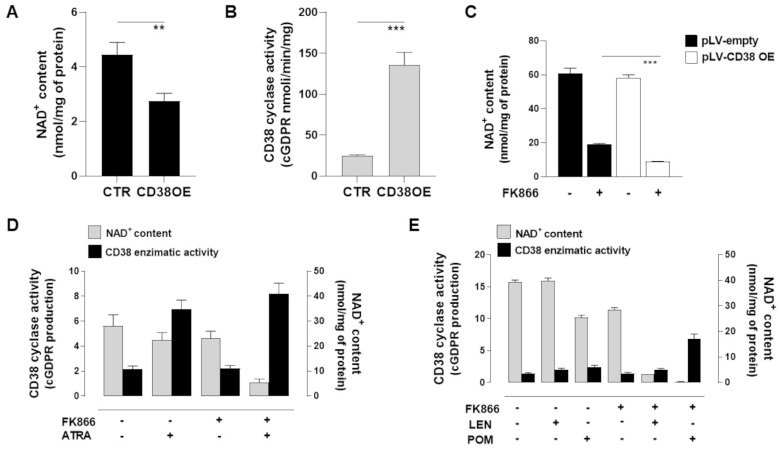
**NAD^+^ depletion accounts for the enhanced sensibility of CD38-upregulated MM cells to NAMPT inhibitor FK866.** (**A**,**B**) LP1 cells infected with lentiviruses overexpressing wild-type CD38 (CD38OE) or empty vector were assayed for their NAD^+^ content (**A**) and CD38 enzymatic activity (GDP-ribosyl cyclase) (**B**). In the same cells, NAD^+^ content (**C**) without FK866 was also measured. Data are presented as mean ± S.D. (*n* = 3). (** *p* ≤ 0.01, *** *p* ≤ 0.001; unpaired *t*-test). (**D**,**E**) Intracellular NAD^+^ level and CD38 enzymatic activity (GDP-ribosyl cyclase) were measured in the H929 cell line after 48 h of treatment with ATRA (1 nM) (**D**), LEN (5 µM), or POM (2.5 µM) (**E**) alone or with FK866 (1 nM).

We previously reported that the NAD^+^-salvage pathway represents an exploitable vulnerability for tumors as the intracellular NAD^+^ depletion triggered by NAMPT-inhibition, resulting in being synergic with several anti-tumor therapies, including anti-MM drugs [11,12,14]. In line with these studies, we here screened metabolic changes triggered by CD38 upregulation at baseline and after exposure to the NAMPT-inhibitor FK866. As expected, CD38-overexpressing MM cells contained lower intracellular NAD^+^ content and higher cGDPR levels than control (Figure 4A,B). In such a scenario, FK866 exposure resulted in being more detrimental to these cells: indeed, intracellular NAD^+^ content depletion triggered by this drug resulted in being more pronounced in CD38-overexpressed cells than the control (Figure 4C). Subsequently, we complemented genetic studies with pharmacological approaches. As shown in Figure 4D,E, CD38-inducing drugs (ATRA, LEN, and POM) sum up earlier results.

### 3.5. NAD^+^ and Nicotinic Acid Abolish Activity of Co-Treatment in Myeloma Cells

**Figure 5:**
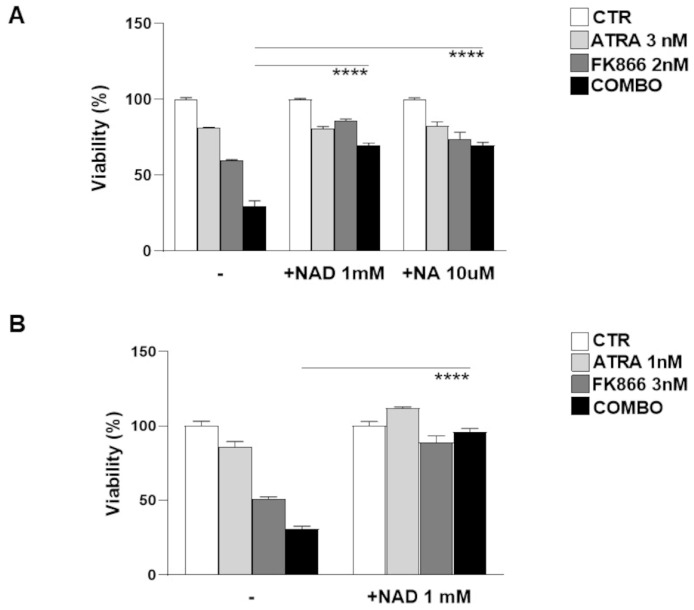
**NAD^+^ and Nicotinic Acid supplementation abolishes the activity of co-treatment in MM cells.** Viability of LP1 (**A**) and H929 (**B**) cells treated as indicated with ATRA (1 or 3 nM), FK866 (2 or 3 nM), and their combination for 96 h in the presence or absence of NAD^+^ (1 mM) or NA (10 µM). Cell viability was measured with MTS assay and presented as a percentage of control. The results are a mean ± SD of triplicate samples (**** *p* ≤ 0.0001; unpaired *t*-test).

Since CD38 modulation markedly enhanced the NAD^+^ depletion triggered by the Nampt inhibitor exposure, we assumed that the energy pool may have had a huge impact on the anti-MM effects of our strategy. As a result, we evaluated the ability of nicotinic acid (NA, the precursor involved in the NAD^+^ biosynthesis) or NAD^+^ (the end product) to abrogate the cell death caused by the tested combination. As shown in Figure 5A,B, supplementation in excess with NAD^+^ and its precursor NA fully abrogated the anti-MM activity of FK866, both as a single agent and in combination with ATRA, confirming the role of NAD^+^ depletion in the activity of tested anti-MM strategy. Overall, these data confirm that the effects observed on MM cell viability are a consequence of a huge energy shortage triggered by these drugs.

### 3.6. Metabolic Reprogramming Elicited by CD38 Overexpression Identifies a Novel Druggable Vulnerability in MM Cells

**Figure 6:**
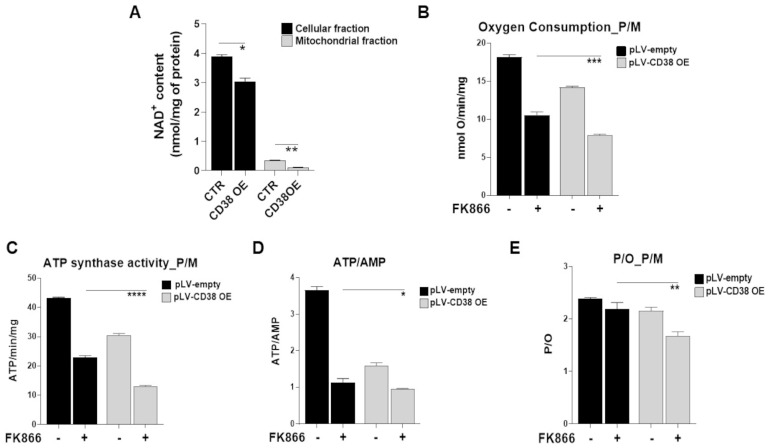
**Metabolic reprogramming elicited by CD38-overexpression identifies a novel druggable vulnerability in MM cells.** (**A**) Cellular and mitochondrial NAD^+^ contents were measured in H929 CD38 OE or control cells and normalized to the protein content of each fraction. (**B**) Oxygen consumption, (**C**) activity of Fo-F1 ATP synthase, (**D**) energy status expressed as ATP/AMP ratio, and (**E**) oxidative phosphorylation efficiency as P/O ratio were measured in H929 control and CD38 OE cells. Data are presented as the mean ± SD of three different experiments. (* *p* ≤ 0.05, ** *p* ≤ 0.01, *** *p* ≤ 0.001, **** *p* ≤ 0.0001; unpaired *t*-test).

Although the cancer cells metabolism is characterized by increased glucose consumption and lactic fermentation (i.e., Warburg effect), mitochondria also play a pivotal role in meeting their energetic and biosynthetic demands [37,38]. While it is well known that preserving the NAD^+^/NADH ratio is essential for mitochondrial function, it is now emerging that the maintenance of the mitochondrial NAD^+^ pool is also critical [39]. As a result, mitochondria and cytosol fractions derived from control and CD38-overexpressing cells were compared for their NAD^+^ content pool. As shown in Figure 6A and Figure S8A, CD38 overexpression resulted in a decrease in whole cellular NAD^+^ content with the mitochondria pool being the most compromised, with their reserves almost gone in this state. These results suggest that a cellular energetic remodeling is triggered by CD38 modulation, which, however, seems to affect mitochondrial NAD^+^ stores to a greater extent. As such, it may be assumed that a different subcellular localization of CD38 could explain the specific energetic impairment of mitochondria in CD38-overexpressing cells.

Previous studies show that NAD^+^-lowering agents affect the OxPhos and cellular energy status of tumor cells [36,40]. Thus, we postulated that an energetic remodeling triggered by NAD^+^-depletion could account for the enhanced vulnerability of CD38-upregulated MM cells to these agents. To address this hypothesis, we measured the oxygen consumption rate (OCR), ATP synthase activity, and ATP/AMP ratio in genetically CD38-overexpressing MM cells in parallel with controls, observing a comprehensive reduction in all these activities (Figure 6B,D and Figure S8B,C). In addition, the remaining OxPhos activity in CD38-overexpressing cells appears partially uncoupled, as shown by P/O values, suggesting a further loss of energy efficiency (Figure 6E). As a consequence of mitochondrial metabolism impairment and energy efficiency loss, the cellular energy status appeared reduced, as indicated by the ATP/AMP, predominantly after FK866 exposure. We next supplemented these data by using CD38-inducing agents LEN, POM, and ATRA: similar to gene handling-derived data, all these stimuli resulted in the comprehensive impairment of respiration capacity rate and energy status, as demonstrated by OCR, ATP production, and ATP/AMP ratio, seen in Figure S9A–C, respectively. Remarkably, a significant reduction of the P/O ratio was also measured with this approach, thus supporting metabolic reprogramming (Figure S9D).

### 3.7. Mitochondrial Dynamic Shift Underlies an Organelle-Specific Dysfunction Triggered by CD38 Upregulation

**Figure 7:**
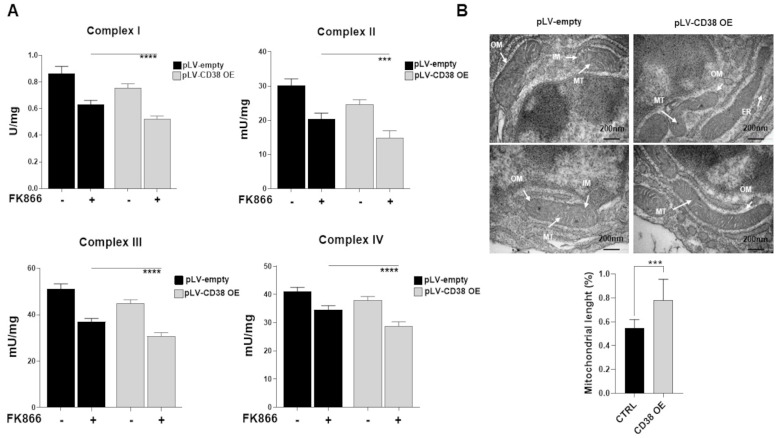
**Mitochondrial dynamic shift underlies an organelle-specific dysfunction triggered by CD38 upregulation**. (**A**) Mitochondrial complexes (I, II, III, IV) activities were measured in H929 control and CD38 OE cells at baseline and following treatment with FK866 (2 nM). (**B**) TEM image of H929 control and CD38 OE cells displaying elongated mitochondria. Scale bars: 200 nm. Average mitochondrial length quantified in μm. Data are presented as mean ± S.D (*n* = 3). (*** *p* ≤ 0.001, **** *p* ≤ 0.0001; unpaired *t*-test).

To obtain insights into the molecular basis of observed metabolic reprogramming, genetically CD38-overexpressing MM cells were analyzed for their total respiratory capacity: as shown in Figure 7A and Figure S10A,B, the measurement of complex I, II, III, and IV activities and protein levels revealed worse capabilities for CD38-modified cells with exposure to FK866, resulting in poorer outcomes. Similar features were observed following CD38-inducer exposure (Figure S11A). As consequence of observed mitochondrial health impairment, CD38-overexpressed cells exhibited higher lactate release (Figure S11B) and glucose uptake (Figure S11C) than control cells, thus suggesting that CD38 upregulation leads to metabolic dysfunction and likely to increased glycolysis dependency in MM cells.

Recently, it has been reported that mitochondria alter their structures as a strategy to optimize metabolic stress induced by different insults [41,42,43]. To this aim, we used electron microscopy analysis to measure mitochondria morphology abnormalities. As shown in Figure 7B, the average mitochondrial length after CD38 overexpression was significantly increased compared with the control along with the appearance of an abnormal mitochondrial morphology, thus confirming an imbalance towards mitochondrial elongation in CD38-upregulated cells. Notably, the observed that mitochondrial morphology changes would in turn represent an attempt by CD38-upregulated cells to face their great energetic stress, as previously reported [44]. Altogether our data suggest that the massive intracellular NAD^+^ depletion produced by CD38 upregulation unmasks the mitochondria machinery alteration as a vulnerability in these cells that could be therapeutically exploited by NAD^+^-lowering agents.

### 3.8. The Oxidative Stress Triggered by Energetic Depletion Is Crucial for Tested Drug Combinations

**Figure 8:**
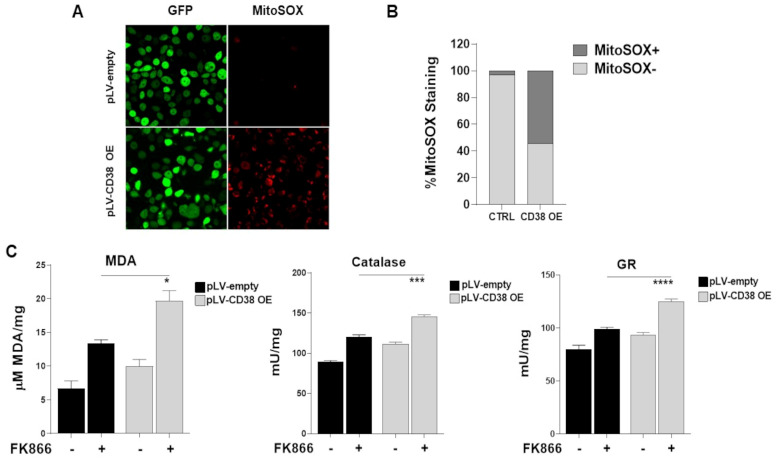
**The oxidative stress triggered by energetic depletion is crucial for tested drugs combination.** Mitochondrial superoxide anions were detected by immunofluorescence (**A**) and flow cytometry (**B**) using MitoSOX, in H929 cells lentivirally transduced with pLVempty vector or pLV CD38 OE. (**C**) Oxidative stress marker (MDA) and activities of antioxidant enzymes (Catalase, Glutathione reductase-GR) were measured in control and CD38 OE cells. Data are presented as mean ± S.D (*n* = 3). (* *p* = 0.05, *** *p* = 0.001, **** *p* ≤ 0.0001; unpaired *t*-test).

The primary function of mitochondria is to support cellular processes by generating energy [45,46]. Indeed, mitochondria constitute a metabolic hub, which produces highly toxic molecules that react with a broad range of metabolites, thus coupling these organelles tightly to ROS generation. In such a scenario, NAMPT inhibitors have been described as producers of oxidative stress at a high level **[47]**. As result, we next investigated whether mitochondrial changes observed in CD38-overexpressing cells shape redox homeostasis as well. In accordance with this assumption, Figure 8A,B and Figure S12A show that CD38 overexpression rapidly increased mitochondrial superoxide anions (MitoSox) in MM cells, which in turn resulted in oxidative damage accumulation, as shown by the increase in malondialdehyde (MDA) and the activation of two enzymes involved in the endogenous antioxidant defenses: catalase and glutathione reductase (Figure 8C and Figure S12B). Similarly, all CD38-inducing pharmacologic approaches recapitulated these events, which were further increased by the addition of a NAD^+^-lowering agent (Figure S13).

**Figure 9:**
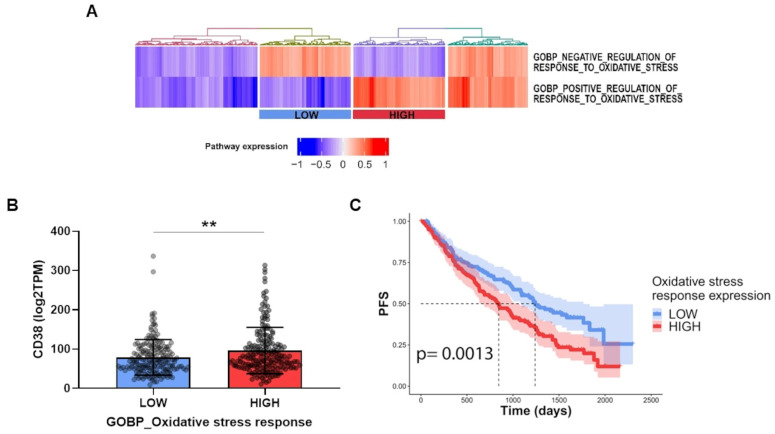
**NAD^+^-depleting agents’ treatment could be beneficial for oxidative stress-prone MM patients.** (**A**) Heatmap displaying enrichment in the CoMMpass dataset of indicated gene ontology terms; “LOW” and “HIGH” rectangles indicate groups of patients with a low and high expression of oxidative stress response, respectively. (**B**) Scatter-bar plot showing CD38 RNA levels in LOW and HIGH groups from panel E. (**C**) Kaplan–Meyer curves of the progression-free survival probability of the LOW and HIGH groups from panel A, *p*-value is indicated. Data are presented as mean ± S.D (*n* = 3). (** *p* ≤ 0.01; unpaired *t*-test).

Collectively, these data suggest that the harmful effects of ROS production are responsible for the anti-MM effects of the tested strategy. To support the idea that a high level of oxidative stress is also the main driver of these events in MM patients, we analyzed RNA sequencing data from the CoMMpass dataset to examine the relevance of CD38 status among oxidative stress-prone tumors. Stratifying samples for their expression of genes involved in the positive and negative regulation of the oxidative stress response, according to gene ontology terms GO:1902884 and GO:1902883, we identified two groups with low and high stress response activation, which mirrored oxidative stress-prone or non-stress prone MM tumors. Importantly, patients with a high stress response demonstrated higher CD38 gene expression levels as well as poor outcomes in terms of progression free survival, regardless of the specific anti-MM-used regimen (Figure 9A–C). Collectively, these data strongly suggest that CD38 upregulation, irrespective of the specific used approach, results in the improved anti-MM activity of NAD^+^-depleting agents, with oxidative stress that appears to be crucial in managing the impact of this strategy among MM patients.

## 4. Discussion

In this study, we have demonstrated that CD38, the major NAD^+^ consumer, regulates the mitochondrial fitness of MM cells and affects their sensibility to NAD^+^-lowering agents. By producing massive intracellular-NAD^+^ shortages, CD38 upregulation results in mitochondrial fitness impairment and metabolic reprogramming, which primes MM cells to NAD^+^-lowering agents. Specifically, genetic or pharmacologic approaches, by increasing CD38 levels, make MM cells more vulnerable to energetic depletion triggered by NAMPT inhibitors. Mechanistically, the abnormal mitochondrial morphology and OxPhos impairment triggered by CD38 upregulation result in metabolic remodeling and oxidative stress enhancement, which in turn support the translational relevance of our strategy.

CD38 is a surface ectoenzyme with NAD^+^ase activity, which is highly expressed on malignant plasma cells: this feature makes it an exploitable target for anti-MM therapies, as demonstrated by the clinical successes of anti-CD38 MoAbs. However, the specific role played by CD38 in MM still remains to be elucidated. Based on its own activity, a consequence of CD38 deregulation is represented by altered intracellular NAD^+^ level maintenance. Specifically, the inhibition of CD38 NAD^+^ase prevents the breakdown of NAD^+^ and thus results in high NAD^+^ levels, which are essential for both normal and cancerous conditions. Based on its role in regulating the myriad of cancer-associated pro-growth biological processes, interrupting NAD^+^ biosynthesis has been proposed as a novel strategy for several tumors, including MM [11,12,48]. Results derived from clinical trials have allowed anti-CD38 MoAbs-based regimens to become the SOC for MM patients’ treatment, both at diagnosis and relapse [15,17,19,23,24,25]. Nevertheless, further steps need to be taken for a full exploitation of CD38-targeting in MM, including a more comprehensive functional analysis of its impact on cell biology. Based on the clinical achievements of anti-CD38 MoAbs, a role for NAD^+^ metabolism is also warranted in MM, thus providing basis for further investigations on NAD^+^ase activity to elucidate the mechanisms of immunotherapy-based approaches. Antigen density on tumor cells is an important determinant for MoAb efficacy, since its loss results in drug-resistance occurrence. As result, several strategies have been found to increase tumor surface CD38, with the goal of boosting MoAb efficacy in co-treatment regimens. Among these agents, ATRA and IMiDs have shown remarkable efficacy in both preclinical and clinical setting [29,33]. Consistent with these data, we found that CD38 inducers result in intracellular NAD^+^ reduction, which primes MM cells for NAD^+^-depleting agents. In contrast, CD38-silencing did not affect the anti-MM activity of these agents, thus indicating energetic depletion as crucial. While some anti-CD38 antibodies do not affect the NAD^+^ase activity of CD38 (e.g., daratumumab), others, such as isatuximab, have been shown to target ectoenzymatic function through the internalization of CD38 [49,50]. Whether NAD^+^ase activity inhibition using an anti-CD38 immunotherapy is beneficial or harmful for tumor cells is still unclear, with studies suggesting a cell-dependent outcome [51,52]. Considering the clinical relevance of anti-CD38-based therapies in MM, we envisage that a combined strategy that aims to upregulate CD38 surface availability to exploit the anti-tumor activity of NAD^+^-depleting agents could be particularly important to eliminate MM cells expressing very low CD38 levels, including low proliferative or dormant MM-initiating cells or drug-resistant clones with minimal residual disease eradication.

Cancer cells continuously adjust energy demand for supporting their growth by increasing the biosynthesis of macromolecules [53]. Against this background, targeting metabolism-related machineries represents a promising tool for anti-cancer therapies, as suggested by the promising results of NAD^+^-lowering agents in solid and blood cancers [54]. Intracellular NAD^+^ levels decrease during aging, together with a parallel increment of CD38 expression and its NAD^+^ase activity [55]. Importantly, these events lead to mitochondrial dysfunction and metabolic abnormalities, thus suggesting a CD38 role in mitochondrial function during aging [56,57,58]. In line with these data, we show that the CD38 upregulation on MM cells results in mitochondrial dysfunction and likely to an increase in glycolysis-dependence. Indeed, mitochondria status analysis revealed an abnormal morphology with a severe loss of NAD^+^ in organelles isolated from CD38-overexpressing cells compared with controls. It is well known that mitochondria alter their structures to tackle metabolic functions impairments triggered by different cellular insults [59]. Mitochondrial dynamics have been recently exploited for anti-tumor strategies, but how this process is regulated still remains elusive with the identification of pathways that control mitochondrial integrity in cancer representing an urgent priority. Here, we demonstrate the critical nature of mitochondrial dynamics in MM cells and suggest how these machineries could be potentially exploited by upregulating CD38 surface levels: we assume that the huge intracellular NAD^+^ depletion triggered by CD38 upregulation unmasks mitochondria machinery dysfunction, thus making it an actionable vulnerability for MM cells. Overall, this proof-of-principle study demonstrates the critical role of mitochondrial dynamics dysfunction in CD38-upregulated activity and supports the use of this approach to prime MM cells through NAD^+^-lowering agents. Hopefully this strategy, by enhancing CD38 surface availability with different approaches, would bring the use of NAD^+^-depleting agents closer to clinical trials involving MM patients.

## 5. Conclusions

**Figure 10:**
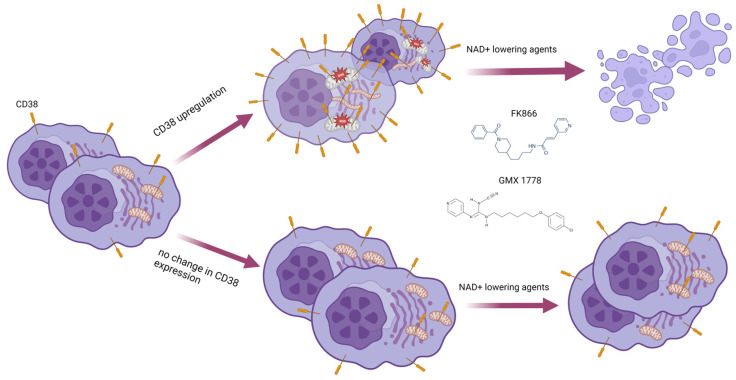
Graphic of proposed model: CD38 upregulation by genetic or pharmacologic (ATRA, LEN, or POM) approaches result in energetic remodeling with mitochondria dynamic shifts and oxidative stress priming MM cells for low doses of NAD^+^-depleting agents.

In summary, by systematically investigating the cellular features of MM cells, here we found that CD38 overexpression affects the energetic status of these cells by shifting mitochondria dynamics and triggering oxidative stress, thus priming the activity of NAD^+^-lowering agents. Specifically, our study shows that CD38 overexpression through different approaches enhances the anti-MM activity of NAMPT inhibitors, with few toxic effects (Figure 10). Overall, therefore, we propose a novel strategy to make MM patients more sensitive to NAD-reducing agents, which, on the basis of their well-established antitumor activity, deserves further clinical development.

## Figures and Tables

**Table 1:** Patients’ characteristics.

Patients	AGE	SEX	ISOTYPE	ISS	R-ISS	Disease Status	FISH	* Immunophenotypic Analysis on Bone Marrow						BMPCs (%)	Biochemical Parameters							Therapy
								CD138	CD38	CD56	CD19	CD20	CD45		Crea (mg/dL)	Hb (mg/dL)	PLT (×10^3^)	WBC (×10^6^)	Beta2 Micro (mg/dL)	LDH (U/L)	Crea (mg/dL)	
MM#1	77	M	IgGk	II	II	RRMM	SR	+	+	+/−	−	+/−	+/−	24	0.8	10.6	232	2.95	3.3	198	DVMP	0.8
MM#2	48	F	IgGk	I	I	NDMM	t(4;14)	+	+	−	+	−	+	30	0.6	13.5	318	13.42	1.3	185	KRD	0.6
MM#3	63	M	IgAk	III	III	NDMM	SR	+	+	+	−	−	+/−	18	1.4	11.5	170	7.22	7	180	KRD	1.4
MM#4	77	M	IgG-l	II	III	RRMM	t(4;14)	+	+	+	−	−	−	19	1.05	9.8	338	12.5	3.78	216	MPV	1.05
MM#5	66	M	IgGk	ND	III	RRMM	t(11;14)	+	+	+	−	−	+	45	1	10	348	9.69	3	340	DVTD	1
MM#6	66	F	Micromolecular	III	III	NDMM	SR	+	++	+	−	−	+/−	25	0.6	8.7	273	4.87	2.2	178	DVTD	0.6
MM#7	85	F	IgGk	II	II	NDMM	del(17p)	++	++	+	−	−	+/−	30	0.8	10.4	237	5.99	3.2	182	MPV	0.8

* Immunophenotypic analysis details are described in supplementary data.

**Table 2:** Genes up-regulated and down-regulated by FK866 treatment in GSE96636, used to build FK866 activity signature.

GENE	SIGNATURE	GENE	SIGNATURE
FLJ32224	FK866dn	SLC30A1	FK866up
LOC100128239	FK866dn	CHD6	FK866up
C4orf47	FK866dn	KIAA1024L	FK866up
PTAFR	FK866dn	PHLDA2	FK866up
CCR5	FK866dn	FLCN	FK866up
LINC00469	FK866dn	LRRC55	FK866up
PON1	FK866dn	PTPN14	FK866up
GLB1L3	FK866dn	VPS13B	FK866up
TMEM132E	FK866dn	POLR2M	FK866up
NPAS2	FK866dn	CSF2	FK866up
DIS3L2	FK866dn	BTBD9	FK866up
MIRLET7BHG	FK866dn	NOM1	FK866up
CFTR	FK866up	LOC647107	FK866up
ZC3HAV1	FK866up	KLHL11	FK866up
NPHS2	FK866up	ZC3H12D	FK866up
MYLIP	FK866up	RASD1	FK866up
P2RX6	FK866up	RGS1	FK866up
FAM22G	FK866up	TAGAP	FK866up

## Data Availability

Multi-omics data of newly diagnosed MM samples were derived from Multiple Myeloma Research Foundation (MMRF) CoMMpass Study and obtained from the Interim Analysis 15a (MMRF_CoMMpass_IA15a).

## Supplementary Materials

The following supporting information can be downloaded at: https://www.mdpi.com/article/10.3390/antiox12020494/s1. Figure S1. Intracellular NAD^+^ content plotted versus CD38 activity in human MM cell lines (HMCLs; N = 11). The Spearman’s rank correlation coefficient (r) and the *p* value was calculated using Graph-Pad Prism software 8.4.3. Figure S2. Indicated HMCLs were lentivirally transduced with pLV empty and pLV CD38 overexpressing (CD38 OE) and then treated with increasing doses of FK866 (0–10 nM) for 96 h. Cell viability was measured with MTS assay and presented as a percentage of control. Data are presented as mean ± S.D (*n* = 3). (** *p* ≤ 0.01, *** *p* ≤ 0.001, **** *p* ≤ 0.0001; unpaired t test). Figure S3. H929 cells were lentivirally transduced with pLV empty and pLV CD38 overexpressing (CD38 OE) and then treated with growing doses of GMX-1777 (0–3 nM) for 96 h. Cell viability was measured with MTS assay and presented as a percentage of control. Data are presented as mean ± S.D (*n* = 3). (** *p* ≤ 0.01, **** *p* ≤ 0.0001; unpaired t test). Figure S4. LP1 cells were incubated with ATRA (ranging from 1 to 0.1 nM) for 24, 48 or 72 h after which cells were collected to analyze CD38 expression levels by flow cytometry. Figure S5. Representative dot plots of MM1S (A) or H929 (B) cells treated with indicated drugs at showed concentration for 96 h. Apoptotic cell death was assessed by flow cytometry using annexin V and propidium iodide double staining by cytofluorimetric analysis. Figure S6. (A,B) LP1 cells were incubated with LEN (A) or POM (B) (ranging from 1 to 0.1 µM) for indicated time points after which cells were collected to analyze CD38 expression levels by flow cytometry. Both panels show fold increase of CD38 median fluorescence intensity (MFI) compared with control. (C,D) Cell viability measured with MTS assay in a H929 cells treated with FK866 (1–1.5 nM), POM (5–10 µM), LEN (5–10 µM) or their combo for 96 h. Displayed are data represented as mean +/− SD in all (*n* = 3). (E) Apoptotic cell death assessed with flow cytometry analysis after Annexin V/PI staining in a panel of HMCLs treated with FK866 (1–3 nM), LEN (0.1–3 µM) or their combo for 96 h. Displayed are data represented as mean +/− SD in all (*n* = 3). (* *p* ≤ 0.05, ** *p* ≤ 0.01, **** *p* ≤ 0.0001; unpaired t test). Figure S7. (A) H929 cells were treated with FK866 (3 nM), ATRA (2 nM) or their combo in the presence or absence of rhIL-6 (10 ng/mL) or rhIGF-1 (100 ng/mL) for 96 h, and cell viability was measured with MTS assay and presented as a percentage of control. (B) H929 cells were treated with FK866 (3 nM), LEN (3 µM) or their combo in the presence or absence of rhIL-6 (10 ng/mL) or rhIGF-1 (100 ng/mL) for 96 h, and cell viability was measured with MTS assay and presented as a percentage of control. The results presented are a mean ± SD of triplicate samples. (**** *p* ≤ 0.0001; unpaired t test). Figure S8. (A) Cellular and mitochondrial NAD^+^ contents were measured in LP1 CD38 OE or control cells and normalized to protein content of each fraction. (B) Oxygen consumption, activity of ATP synthase and ATP/AMP ratio (C) were measured in LP1 CD38 OE or control cells. Data are presented as mean ± SD of three different experiments. (* *p* ≤ 0.05, ** *p* ≤ 0.01, *** *p* ≤ 0.001; unpaired t test). Figure S9. (A) Oxygen consumption, (B) activity of Fo-F1 ATP synthase, (C) energy status expressed as ATP/AMP ratio and P/O ratio (D), were measured in H929 treated with FK866 (2 nM), ATRA (3 nM), Len (3 µM) and their combination for 96 h. Data are presented as mean ± SD of three different experiments. (* *p* ≤ 0.05, ** *p* ≤ 0.01, *** *p* ≤ 0.001; unpaired t test). Figure S10. (A) Mitochondrial complexes (I, II, III, IV) activities were measured in LP1 control and CD38 OE cells at baseline. (B) Mitochondrial complex II expression was also evaluated by western blot analysis: CD38 overexpression decreases mitochondrial complex marker SDHB (complex II) compared with control cells. Data are presented as mean ± SD of three different experiments. (**** *p* ≤ 0.0001; unpaired t test). Figure S11. (A) Mitochondrial complexes (I, II, III, IV) activities were measured in H929 cells following treatment with FK866 (2 nM), ATRA (3 nM), Len (3 µM) or their combos for 96 h. (B) Lactate and (C) glucose levels were measured in indicated cell lines infected with lentiviruses carrying a wild-type version of CD38 (CD38OE) or empty vector. Data are presented as mean ± S.D (*n* = 3). (* *p* ≤ 0.05, ** *p* ≤ 0.01, *** *p* ≤ 0.001, **** *p* ≤ 0.0001; unpaired t test). Figure S12. (A) Mitochondrial superoxide anions were detected by flow cytometry using MitoSOX, in LP1 cells lentivirally transduced with pLVempty vector or pLV CD38 OE. (B) Oxidative stress marker (MDA) and activities of antioxidant enzymes (Catalase and Glutathione reductase-GR) were measured in LP1 cells lentivirally transduced with pLVempty vector or pLV CD38 OE. Data are ± SD, *n* = 3. (**** *p* ≤ 0.0001; unpaired t test). Figure S13. Oxidative stress marker (MDA) and activities of antioxidant enzymes (Catalase, Glutathione reductase-GR) were measured in CD38 inducers (ATRA or Len)-treated cells. Data are presented as mean ± S.D (*n* = 3). (** *p* ≤ 0.01, *** *p* = 0.001, **** *p* ≤ 0.0001; unpaired t test). References [60,61,62,63,64,65] are cited in the supplementary materials.

## References

[1] Kyle R.A., Rajkumar S.V. (2004). Multiple Myeloma. N. Engl. J. Med..

[2] Palumbo A., Anderson K. (2011). Multiple Myeloma. N. Engl. J. Med..

[3] Mikhael J., Ismaila N., Cheung M.C., Costello C., Dhodapkar M.V., Kumar S., Lacy M., Lipe B., Little R.F., Nikonova A. (2019). Treatment of Multiple Myeloma: ASCO and CCO Joint Clinical Practice Guideline. J. Clin. Oncol..

[4] Hanahan D., Weinberg R.A. (2000). The Hallmarks of Cancer. Cell.

[5] Lunt S.Y., Vander Heiden M.G. (2011). Aerobic Glycolysis: Meeting the Metabolic Requirements of Cell Proliferation. Annu. Rev. Cell Dev. Biol..

[6] Yaku K., Okabe K., Hikosaka K., Nakagawa T. (2018). NAD Metabolism in Cancer Therapeutics. Front. Oncol..

[7] Navas L.E., Carnero A. (2021). NAD^+^ metabolism, stemness, the immune response, and cancer. Signal Transduct. Target. Ther..

[8] Galli U., Travelli C., Massarotti A., Fakhfouri G., Rahimian R., Tron G.C., Genazzani A.A. (2013). Medicinal Chemistry of Nicotinamide Phosphoribosyltransferase (NAMPT) Inhibitors. J. Med. Chem..

[9] Montecucco F., Cea M., Cagnetta A., Damonte P., Nahimana A., Ballestrero A., Rio A., Bruzzone S., Nencioni A. (2013). Nicotinamide Phosphoribosyltransferase as a Target in Inflammation- Related Disorders. Curr. Top. Med. Chem..

[10] Shackelford R.E., Mayhall K., Maxwell N.M., Kandil E., Coppola D. (2013). Nicotinamide Phosphoribosyltransferase in Malignancy: A Review. Genes Cancer.

[11] Cea M., Cagnetta A., Fulciniti M., Tai Y.T., Hideshima T., Chauhan D., Roccaro A., Sacco A., Calimeri T., Cottini F. (2012). Targeting NAD^+^ salvage pathway induces autophagy in multiple myeloma cells via mTORC1 and extracellular signal-regulated kinase (ERK1/2) inhibition. Blood.

[12] Cagnetta A., Cea M., Calimeri T., Acharya C., Fulciniti M., Tai Y.-T., Hideshima T., Chauhan D., Zhong M.Y., Patrone F. (2013). Intracellular NAD^+^ depletion enhances bortezomib-induced anti-myeloma activity. Blood.

[13] Cagnetta A., Caffa I., Acharya C., Soncini D., Acharya P., Adamia S., Pierri I., Bergamaschi M., Garuti A., Fraternali G. (2015). APO866 Increases Antitumor Activity of Cyclosporin-A by Inducing Mitochondrial and Endoplasmic Reticulum Stress in Leukemia Cells. Clin. Cancer Res..

[14] Cea M., Cagnetta A., Acharya C., Acharya P., Tai Y.-T., Yang C., Lovera D., Soncini D., Miglino M., Fraternali-Orcioni G. (2016). Dual NAMPT and BTK Targeting Leads to Synergistic Killing of Waldenström Macroglobulinemia Cells Regardless of MYD88 and CXCR4 Somatic Mutation Status. Clin. Cancer Res..

[15] Dimopoulos M.A., Oriol A., Nahi H., San-Miguel J., Bahlis N.J., Usmani S.Z., Rabin N., Orlowski R.Z., Komarnicki M., Suzuki K. (2016). Daratumumab, Lenalidomide, and Dexamethasone for Multiple Myeloma. N. Engl. J. Med..

[16] Palumbo A., Chanan-Khan A., Weisel K., Nooka A.K., Masszi T., Beksac M., Spicka I., Hungria V., Munder M., Mateos M.V. (2016). Daratumumab, Bortezomib, and Dexamethasone for Multiple Myeloma. N. Engl. J. Med..

[17] Facon T., Kumar S., Plesner T., Orlowski R.Z., Moreau P., Bahlis N., Basu S., Nahi H., Hulin C., Quach H. (2019). Daratumumab plus Lenalidomide and Dexamethasone for Untreated Myeloma. N. Engl. J. Med..

[18] Dimopoulos M.A., Terpos E., Boccadoro M., Delimpasi S., Beksac M., Katodritou E., Moreau P., Baldini L., Symeonidis A., Bila J. (2021). Daratumumab plus pomalidomide and dexamethasone versus pomalidomide and dexamethasone alone in previously treated multiple myeloma (APOLLO): An open-label, randomised, phase 3 trial. Lancet Oncol..

[19] Mateos M.-V., Cavo M., Blade J., Dimopoulos M.A., Suzuki K., Jakubowiak A., Knop S., Doyen C., Lucio P., Nagy Z. (2020). Overall survival with daratumumab, bortezomib, melphalan, and prednisone in newly diagnosed multiple myeloma (ALCYONE): A randomised, open-label, phase 3 trial. Lancet.

[20] Moreau P., Hulin C., Perrot A., Arnulf B., Belhadj K., Benboubker L., Béné M.C., Zweegman S., Caillon H., Caillot D. (2021). Maintenance with daratumumab or observation following treatment with bortezomib, thalidomide, and dexamethasone with or without daratumumab and autologous stem-cell transplant in patients with newly diagnosed multiple myeloma (CASSIOPEIA): An open-label. Lancet Oncol..

[21] Bruzzone S., Moreschi I., Usai C., Guida L., Damonte G., Salis A., Scarfì S., Millo E., De Flora A., Zocchi E. (2007). Abscisic acid is an endogenous cytokine in human granulocytes with cyclic ADP-ribose as second messenger. Proc. Natl. Acad. Sci. USA.

[22] Bruzzone S., Fruscione F., Morando S., Ferrando T., Poggi A., Garuti A., D’Urso A., Selmo M., Benvenuto F., Cea M. (2009). Catastrophic NAD^+^ Depletion in Activated T Lymphocytes through Nampt Inhibition Reduces Demyelination and Disability in EAE. PLoS ONE.

[23] Yamaguchi R., Andreyev A., Murphy A.N., Perkins G.A., Ellisman M.H., Newmeyer D.D. (2007). Mitochondria frozen with trehalose retain a number of biological functions and preserve outer membrane integrity. Cell Death Differ..

[24] Cagnetta A., Soncini D., Orecchioni S., Talarico G., Minetto P., Guolo F., Retali V., Colombo N., Carminati E., Clavio M. (2018). Depletion of SIRT6 enzymatic activity increases acute myeloid leukemia cells’ vulnerability to DNA-damaging agents. Haematologica.

[25] Moreau P., Attal M., Hulin C., Arnulf B., Belhadj K., Benboubker L., Béné M.C., Broijl A., Caillon H., Caillot D. (2019). Bortezomib, thalidomide, and dexamethasone with or without daratumumab before and after autologous stem-cell transplantation for newly diagnosed multiple myeloma (CASSIOPEIA): A randomised, open-label, phase 3 study. Lancet.

[26] Dimopoulos M., Quach H., Mateos M.-V., Landgren O., Leleu X., Siegel D., Weisel K., Yang H., Klippel Z., Zahlten-Kumeli A. (2020). Carfilzomib, dexamethasone, and daratumumab versus carfilzomib and dexamethasone for patients with relapsed or refractory multiple myeloma (CANDOR): Results from a randomised, multicentre, open-label, phase 3 study. Lancet.

[27] Moreau P., Dimopoulos M.-A., Mikhael J., Yong K., Capra M., Facon T., Hajek R., Špička I., Baker R., Kim K. (2021). Isatuximab, carfilzomib, and dexamethasone in relapsed multiple myeloma (IKEMA): A multicentre, open-label, randomised phase 3 trial. Lancet.

[28] Malavasi F., Deaglio S., Funaro A., Ferrero E., Horenstein A.L., Ortolan E., Vaisitti T., Aydin S. (2008). Evolution and Function of the ADP Ribosyl Cyclase/CD38 Gene Family in Physiology and Pathology. Physiol. Rev..

[29] Nijhof I.S., Groen R.W.J., Lokhorst H.M., van Kessel B., Bloem A.C., van Velzen J., de Jong-Korlaar R., Yuan H., Noort W.A., Klein S.K. (2015). Upregulation of CD38 expression on multiple myeloma cells by all-trans retinoic acid improves the efficacy of daratumumab. Leukemia.

[30] García-Guerrero E., Gogishvili T., Danhof S., Schreder M., Pallaud C., Pérez-Simón J.A., Einsele H., Hudecek M. (2017). Panobinostat induces CD38 upregulation and augments the antimyeloma efficacy of daratumumab. Blood.

[31] Choudhry P., Mariano M.C., Geng H., Martin T.G., Wolf J.L., Wong S.W., Shah N., Wiita A.P. (2020). DNA methyltransferase inhibitors upregulate CD38 protein expression and enhance daratumumab efficacy in multiple myeloma. Leukemia.

[32] Xing L., Wang S., Liu J., Yu T., Chen H., Wen K., Li Y., Lin L., Hsieh P.A., Cho S.-F. (2021). BCMA-Specific ADC MEDI2228 and Daratumumab Induce Synergistic Myeloma Cytotoxicity via IFN-Driven Immune Responses and Enhanced CD38 Expression. Clin. Cancer Res..

[33] Fedele P.L., Willis S.N., Liao Y., Low M.S., Rautela J., Segal D.H., Gong J.-N., Huntington N.D., Shi W., Huang D.C.S. (2018). IMiDs prime myeloma cells for daratumumab-mediated cytotoxicity through loss of Ikaros and Aiolos. Blood.

[34] Ogiya D., Liu J., Ohguchi H., Kurata K., Samur M.K., Tai Y.-T., Adamia S., Ando K., Hideshima T., Anderson K.C. (2020). The JAK-STAT pathway regulates CD38 on myeloma cells in the bone marrow microenvironment: Therapeutic implications. Blood.

[35] García-Guerrero E., Götz R., Doose S., Sauer M., Rodríguez-Gil A., Nerreter T., Kortüm K.M., Pérez-Simón J.A., Einsele H., Hudecek M. (2021). Upregulation of CD38 expression on multiple myeloma cells by novel HDAC6 inhibitors is a class effect and augments the efficacy of daratumumab. Leukemia.

[36] Thongon N., Zucal C., D’Agostino V.G., Tebaldi T., Ravera S., Zamporlini F., Piacente F., Moschoi R., Raffaelli N., Quattrone A. (2018). Cancer cell metabolic plasticity allows resistance to NAMPT inhibition but invariably induces dependence on LDHA. Cancer Metab..

[37] Spinelli J.B., Haigis M.C. (2018). The multifaceted contributions of mitochondria to cellular metabolism. Nat. Cell Biol..

[38] Chakrabarty R.P., Chandel N.S. (2021). Mitochondria as Signaling Organelles Control Mammalian Stem Cell Fate. Cell Stem Cell.

[39] Stein L.R., Imai S. (2012). The dynamic regulation of NAD metabolism in mitochondria. Trends Endocrinol. Metab..

[40] Zucal C., D’Agostino V.G., Casini A., Mantelli B., Thongon N., Soncini D., Caffa I., Cea M., Ballestrero A., Quattrone A. (2015). EIF2A-dependent translational arrest protects leukemia cells from the energetic stress induced by NAMPT inhibition. BMC Cancer.

[41] Chen H., Chan D.C. (2017). Mitochondrial Dynamics in Regulating the Unique Phenotypes of Cancer and Stem Cells. Cell Metab..

[42] Youle R.J., van der Bliek A.M. (2012). Mitochondrial Fission, Fusion, and Stress. Science.

[43] Chen H., McCaffery J.M., Chan D.C. (2007). Mitochondrial Fusion Protects against Neurodegeneration in the Cerebellum. Cell.

[44] Gomes L.C., Di Benedetto G., Scorrano L. (2011). During autophagy mitochondria elongate, are spared from degradation and sustain cell viability. Nat. Cell Biol..

[45] Gammella E., Recalcati S., Cairo G. (2016). Dual Role of ROS as Signal and Stress Agents: Iron Tips the Balance in favor of Toxic Effects. Oxid. Med. Cell. Longev..

[46] Ježek J., Cooper K., Strich R. (2018). Reactive Oxygen Species and Mitochondrial Dynamics: The Yin and Yang of Mitochondrial Dysfunction and Cancer Progression. Antioxidants.

[47] Cloux A.-J., Aubry D., Heulot M., Widmann C., ElMokh O., Piacente F., Cea M., Nencioni A., Bellotti A., Bouzourène K. (2019). Reactive oxygen/nitrogen species contribute substantially to the antileukemia effect of APO866, a NAD lowering agent. Oncotarget.

[48] Cea M., Cagnetta A., Adamia S., Acharya C., Tai Y.-T.T., Fulciniti M., Ohguchi H., Munshi A., Acharya P., Bhasin M.K. (2016). Evidence for a role of the histone deacetylase SIRT6 in DNA damage response of multiple myeloma cells. Blood.

[49] Moreno L., Perez C., Zabaleta A., Manrique I., Alignani D., Ajona D., Blanco L., Lasa M., Maiso P., Rodriguez I. (2019). The Mechanism of Action of the Anti-CD38 Monoclonal Antibody Isatuximab in Multiple Myeloma. Clin. Cancer Res..

[50] Deckert J., Wetzel M.-C., Bartle L.M., Skaletskaya A., Goldmacher V.S., Vallée F., Zhou-Liu Q., Ferrari P., Pouzieux S., Lahoute C. (2014). SAR650984, A Novel Humanized CD38-Targeting Antibody, Demonstrates Potent Antitumor Activity in Models of Multiple Myeloma and Other CD38+ Hematologic Malignancies. Clin. Cancer Res..

[51] Chmielewski J.P., Bowlby S.C., Wheeler F.B., Shi L., Sui G., Davis A.L., Howard T.D., D’Agostino R.B., Miller L.D., Sirintrapun S.J. (2018). CD38 Inhibits Prostate Cancer Metabolism and Proliferation by Reducing Cellular NAD^+^ Pools. Mol. Cancer Res..

[52] Chini C.C.S., Guerrico A.M.G., Nin V., Camacho-Pereira J., Escande C., Barbosa M.T., Chini E.N. (2014). Targeting of NAD Metabolism in Pancreatic Cancer Cells: Potential Novel Therapy for Pancreatic Tumors. Clin. Cancer Res..

[53] Vander Heiden M., Cantley L., Thompson C. (2009). Understanding the Warburg effect: The metabolic requirements of cell proliferation. Science.

[54] Ghanem M.S., Monacelli F., Nencioni A. (2021). Advances in NAD-Lowering Agents for Cancer Treatment. Nutrients.

[55] Camacho-Pereira J., Tarragó M.G., Chini C.C.S., Nin V., Escande C., Warner G.M., Puranik A.S., Schoon R.A., Reid J.M., Galina A. (2016). CD38 Dictates Age-Related NAD Decline and Mitochondrial Dysfunction through an SIRT3-Dependent Mechanism. Cell Metab..

[56] Zhu X.-H., Lu M., Lee B.-Y., Ugurbil K., Chen W. (2015). In vivo NAD assay reveals the intracellular NAD contents and redox state in healthy human brain and their age dependences. Proc. Natl. Acad. Sci. USA.

[57] Gomes A.P., Price N.L., Ling A.J.Y., Moslehi J.J., Montgomery M.K., Rajman L., White J.P., Teodoro J.S., Wrann C.D., Hubbard B.P. (2013). Declining NAD^+^ Induces a Pseudohypoxic State Disrupting Nuclear-Mitochondrial Communication during Aging. Cell.

[58] Scheibye-Knudsen M., Mitchell S.J., Fang E.F., Iyama T., Ward T., Wang J., Dunn C.A., Singh N., Veith S., Hasan-Olive M.M. (2014). A High-Fat Diet and NAD + Activate Sirt1 to Rescue Premature Aging in Cockayne Syndrome. Cell Metab..

[59] Abeliovich H., Zarei M., Rigbolt K.T.G., Youle R.J., Dengjel J. (2013). Involvement of mitochondrial dynamics in the segregation of mitochondrial matrix proteins during stationary phase mitophagy. Nat. Commun..

[60] Soncini D., Orecchioni S., Ruberti S., Minetto P., Martinuzzi C., Agnelli L., Todoerti K., Cagnetta A., Miglino M., Clavio M. (2020). The new small tyrosine kinase inhibitor ARQ531 targets acute myeloid leukemia cells by disrupting multiple tumor-addicted programs. Haematologica.

[61] Soncini D., Minetto P., Martinuzzi C., Becherini P., Fenu V., Guolo F., Todoerti K., Calice G., Contini P., Miglino M. (2020). Amino acid depletion triggered by ʟ-asparaginase sensitizes MM cells to carfilzomib by inducing mitochondria ROS-mediated cell death. Blood Adv..

[62] Marini C., Ravera S., Buschiazzo A., Bianchi G., Orengo A.M., Bruno S., Bottoni G., Emionite L., Pastorino F., Monteverde E. (2016). Discovery of a novel glucose metabolism in cancer: The role of endoplasmic reticulum beyond glycolysis and pentose phosphate shunt. Sci. Rep..

[63] Ravera S., Aluigi M.G., Calzia D., Ramoino P., Morelli A., Panfoli I. (2011). Evidence for ectopic aerobic ATP production on C6 glioma cell plasma membrane. Cell. Mol. Neurobiol..

[64] Bartolucci M., Ravera S., Garbarino G., Ramoino P., Ferrando S., Calzia D., Candiani S., Morelli A., Panfoli I. (2015). Functional Expression of Electron Transport Chain and FoF1-ATP Synthase in Optic Nerve Myelin Sheath. Neurochem. Res..

[65] Picard M., White K., Turnbull D.M. (2013). Mitochondrial morphology, topology, and membrane interactions in skeletal muscle: A quantitative three-dimensional electron microscopy study. J. Appl. Physiol..

