# Supplementary material for: CD38-Induced Metabolic Dysfunction Primes Multiple Myeloma Cells for NAD+-Lowering Agents
Source: Antioxidants (Basel). 2023 Feb 15;12(2):494. doi: 10.3390/antiox12020494 (PMC9952390; doi:10.3390/antiox12020494)
Supplement: Supplementary file 1 [file antioxidants-12-00494-s001.zip › Supplementary Figures.pdf]

Supplementary Figures

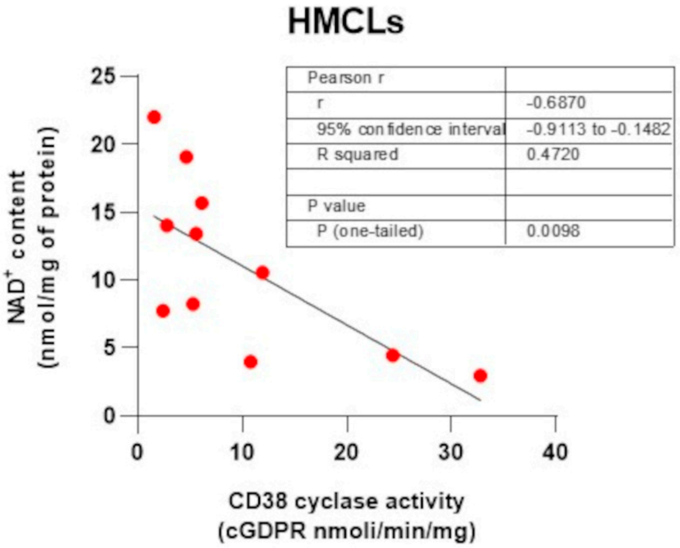

Figure S1. Intracellular NAD^+ content plotted versus CD38 activity in human MM cell lines (HMCLs; N=11). The Spearman's rank correlation coefficient (r) and the p value was calculated using Graph-Pad Prism software 8.4.3.

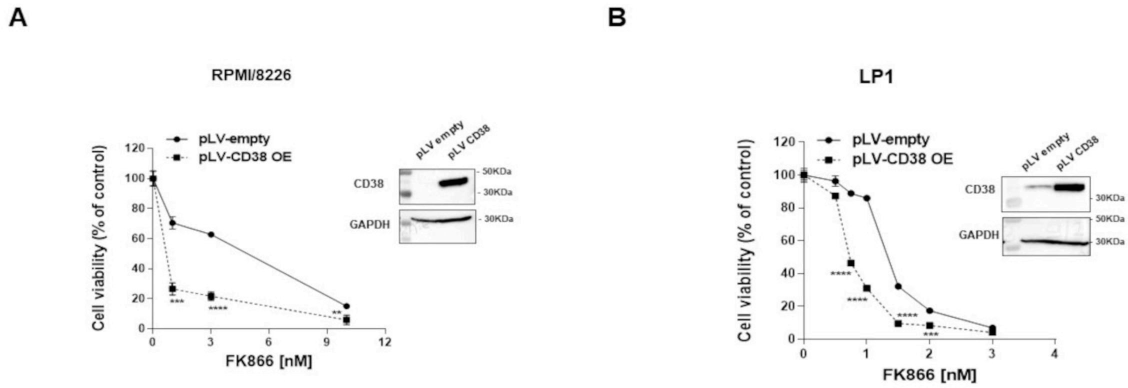

Figure S2. Indicated HMCLs were lentivirally transduced with pLV empty and pLV CD38 overexpressing (CD38 OE) and then treated with increasing doses of FK866 (0-10nM) for 96h. Cell viability was measured with MTS assay and presented as a percentage of control. Data are presented as mean ± S.D (n=3). (**p≤0.01, *** p ≤ 0.001, **** p ≤ 0.0001; unpaired t test).

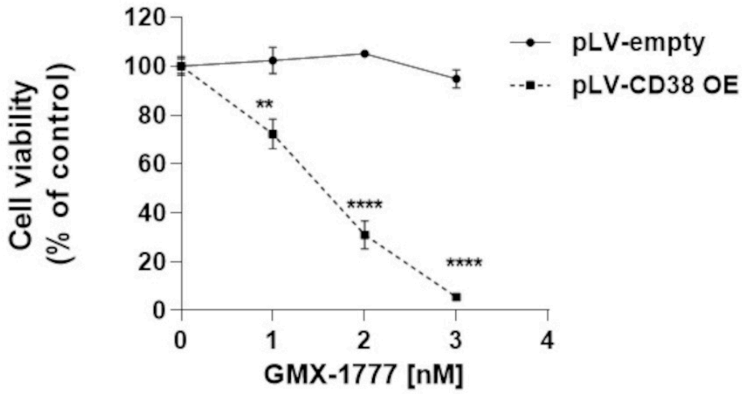

Figure S3. H929 cells were lentivirally transduced with pLV empty and pLV CD38 overexpressing (CD38 OE) and then treated with growing doses of GMX-1777 (0-3nM) for 96h. Cell viability was measured with MTS assay and presented as a percentage of control. Data are presented as mean ± S.D (n=3). (**p≤0.01, **** p ≤ 0.0001; unpaired t test).

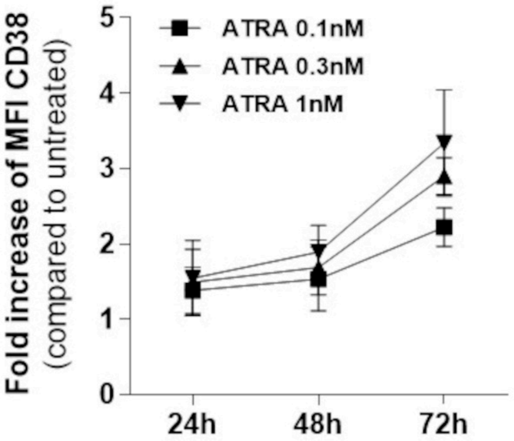

Figure S4. LP1 cells were incubated with ATRA (ranging from 1 to 0.1 nM) for 24, 48 or 72 h after which cells were collected to analyze CD38 expression levels by flow cytometry.

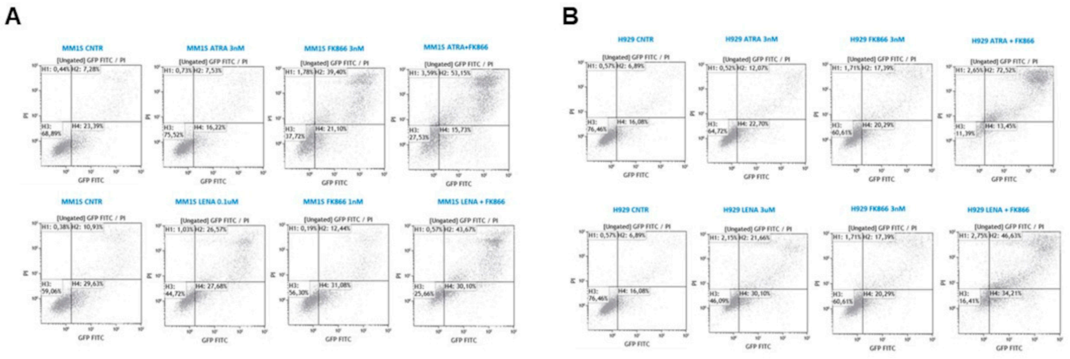

Figure S5. Representative dot plots of MM1S **(A)** or H929 **(B)** cells treated with indicated drugs at showed concentration for 96 hours. Apoptotic cell death was assessed by flow cytometry using annexin V and propidium iodide double staining by cytofluorimetric analysis.

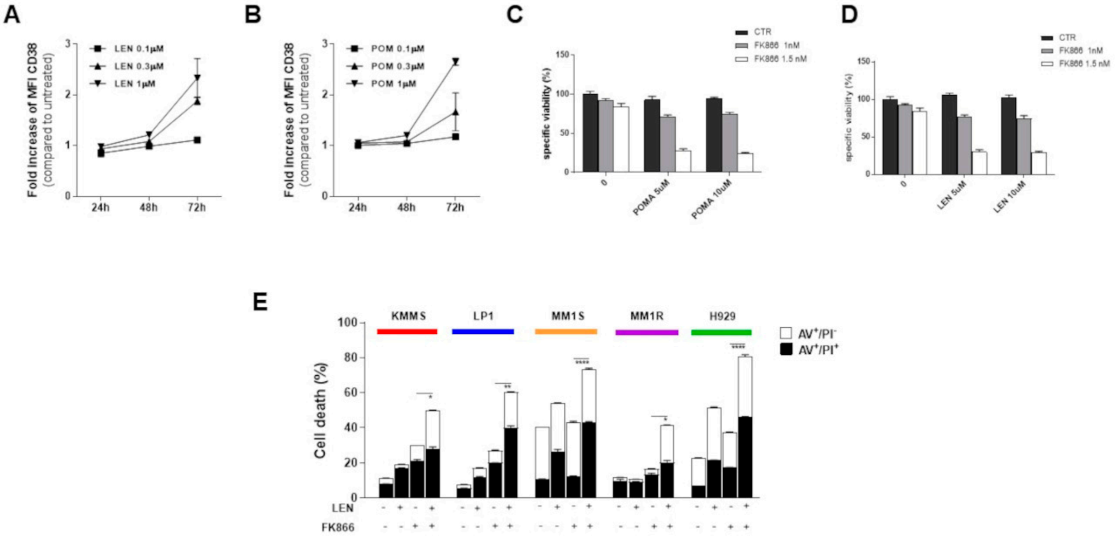

Figure S6. A, B) LP1 cells were incubated with LEN (**A**) or POM (**B**) (ranging from 1 to 0.1 µM) for indicated time points after which cells were collected to analyze CD38 expression levels by flow cytometry. Both panels show fold increase of CD38 median fluorescence intensity (MFI) compared with control. **C-D)** Cell viability measured with MTS assay in a H929 cells treated with FK866 (1-1.5 nM), POM (5-10 µM), LEN (5-10 µM) or their combo for 96h. Displayed are data represented as mean +/− SD in all (n = 3). **E)** Apoptotic cell death assessed with flow cytometry analysis after Annexin V/PI staining in a panel of HMCLs treated with FK866 (1-3 nM), LEN (0.1-3 µM) or their combo for 96h. Displayed are data represented as mean +/− SD in all (n = 3). (*p≤0.05, **p≤0.01, **** p ≤ 0.0001; unpaired t test).

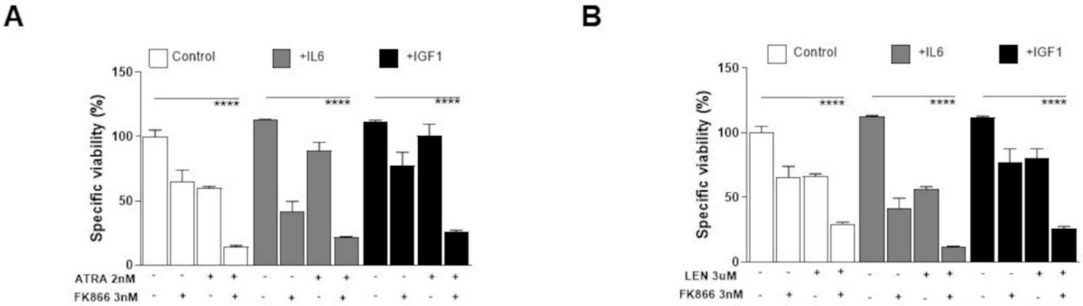

Figure S7. A) H929 cells were treated with FK866 (3 nM), ATRA (2 nM) or their combo in the presence or absence of rhIL-6 (10 ng/mL) or rhIGF-1 (100 ng/mL) for 96 h, and cell viability was measured with MTS assay and presented as a percentage of control. **B)** H929 cells were treated with FK866 (3 nM), LEN (3 μM) or their combo in the presence or absence of rhIL-6 (10 ng/mL) or rhIGF-1 (100 ng/mL) for 96h, and cell viability was measured with MTS assay and presented as a percentage of control. The results presented are a mean ± SD of triplicate samples. (**** p ≤ 0.0001; unpaired t test).

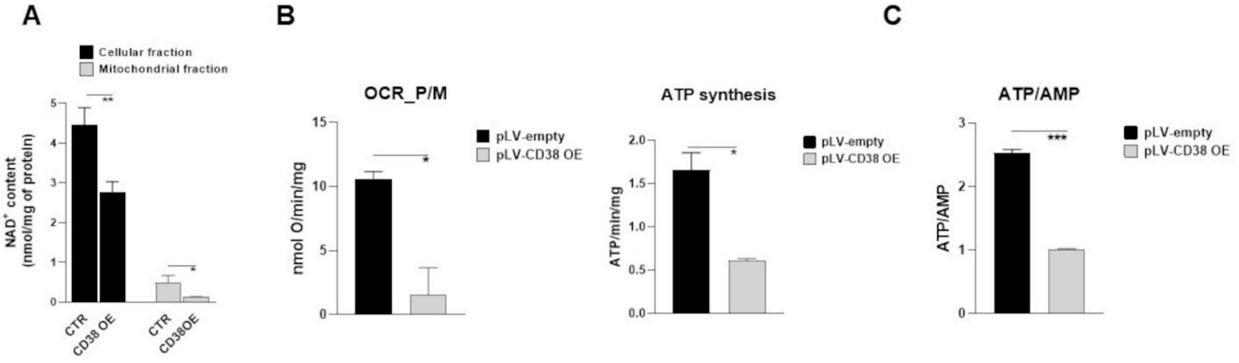

Figure S8. A) Cellular and mitochondrial NAD^+ contents were measured in LP1 CD38 OE or control cells and normalized to protein content of each fraction. **B**) Oxygen consumption, activity of ATP synthase and ATP/AMP ratio **(C)** were measured in LP1 CD38 OE or control cells. Data are presented as mean ± SD of three different experiments. (*p≤0.05, **p≤0.01, *** p ≤ 0.001; unpaired t test).

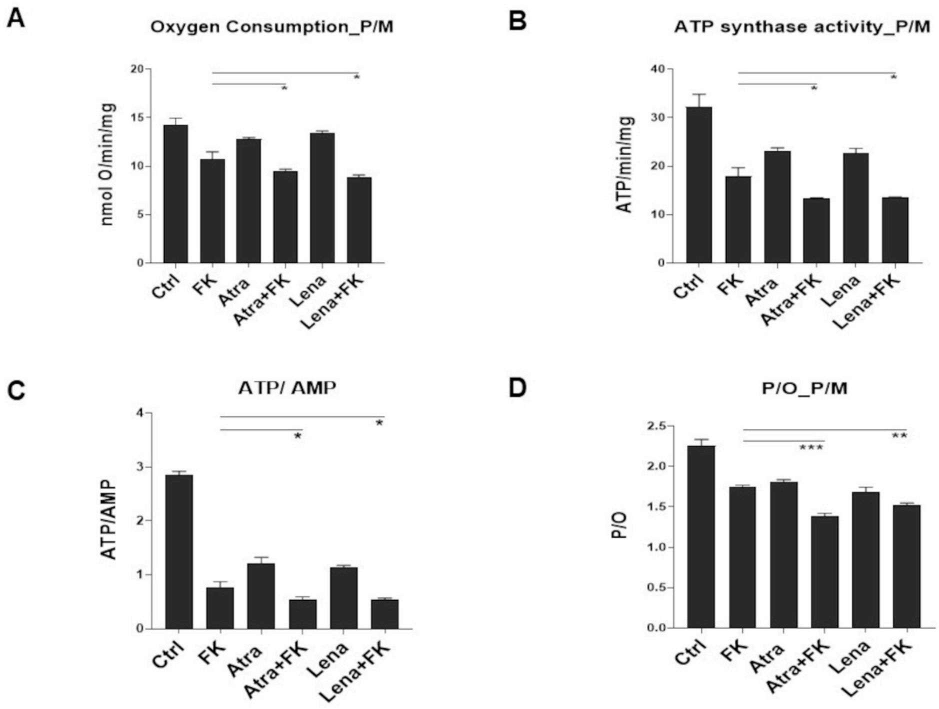

Figure S9. A) Oxygen consumption, **(B)** activity of Fo-F1 ATP synthase, **(C)** energy status expressed as ATP/AMP ratio and P/O ratio **(D)**, were measured in H929 treated with FK866 (2 nM), ATRA (3 nM), Len (3 μM) and their combination for 96h. Data are presented as mean ± SD of three different experiments. (*p≤0.05, **p≤0.01, *** p ≤ 0.001; unpaired t test).

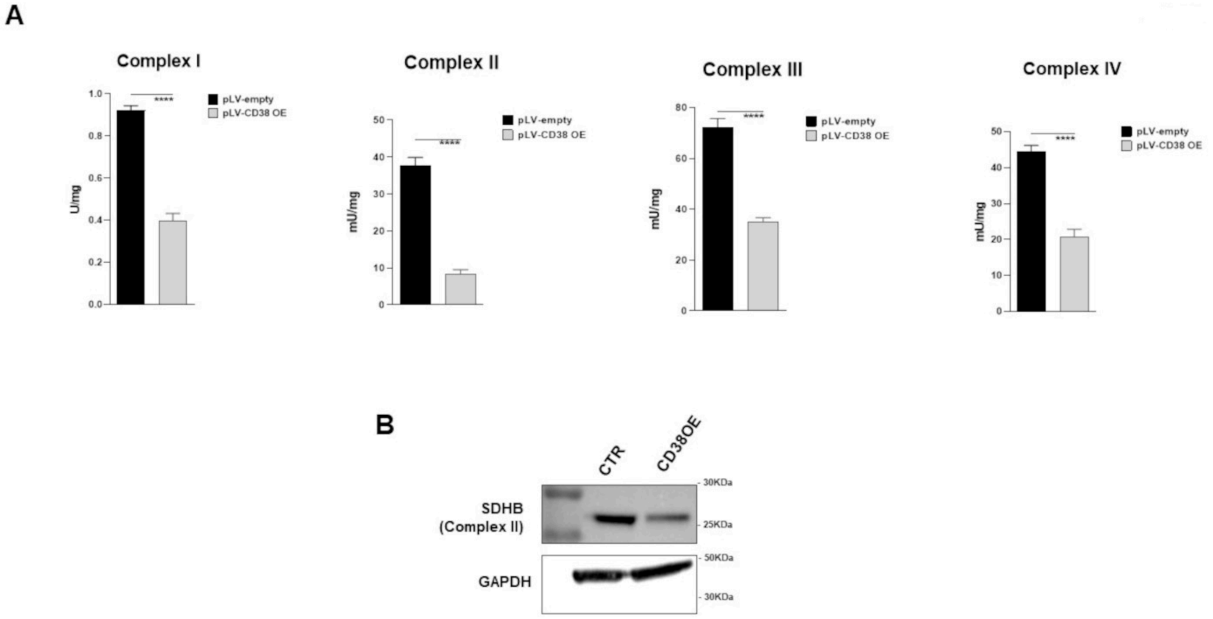

Figure S10. A) Mitochondrial complexes (I, II, III, IV) activities were measured in LP1 control and CD38 OE cells at baseline. **B)** Mitochondrial complex II expression was also evaluated by western

blot analysis: CD38 overexpression decreases mitochondrial complex marker SDHB (complex II) compared with control cells. Data are presented as mean ± SD of three different experiments. (**** p ≤ 0.0001; unpaired t test).

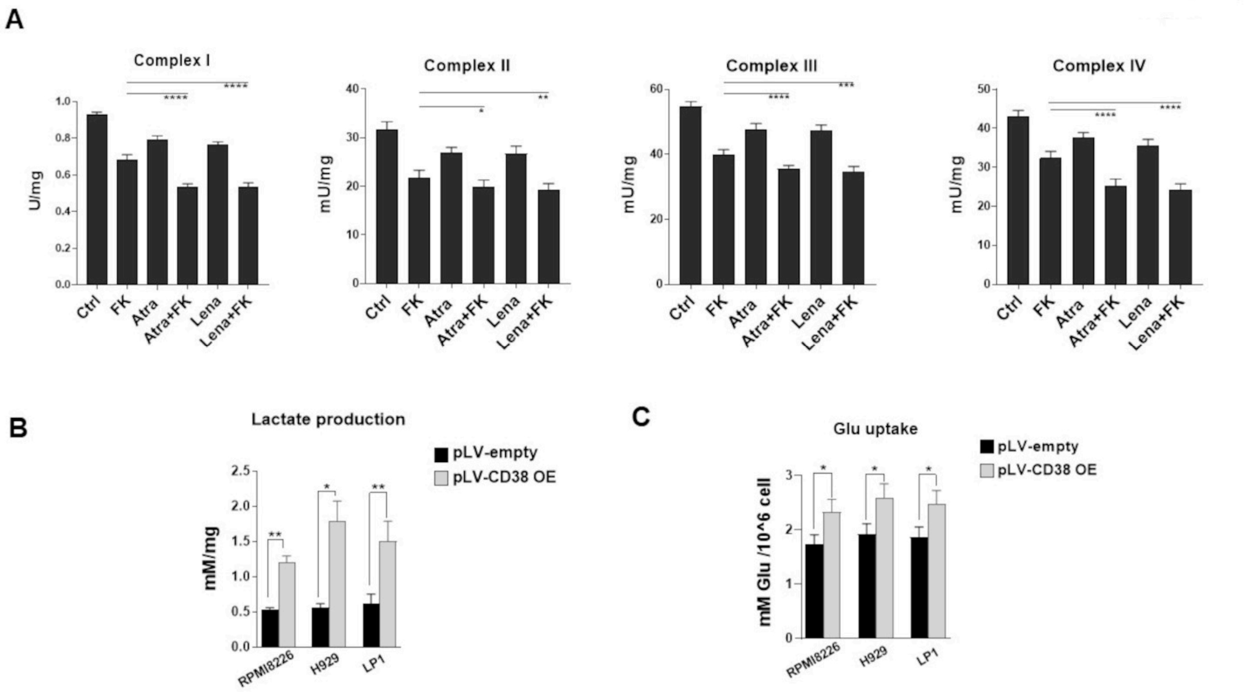

Figure S11. A) Mitochondrial complexes (I, II, III, IV) activities were measured in H929 cells following treatment with FK866 (2 nM), ATRA (3 nM), Len (3 μM) or their combos for 96h. **B)** Lactate and **C)** glucose levels were measured in indicated cell lines infected with lentiviruses carrying a wild-type version of CD38 (CD38OE) or empty vector. Data are presented as mean ± S.D (n=3). (*p⩽0.05, **p⩽0.01, *** p ⩽ 0.001, **** p ⩽ 0.0001; unpaired t test).

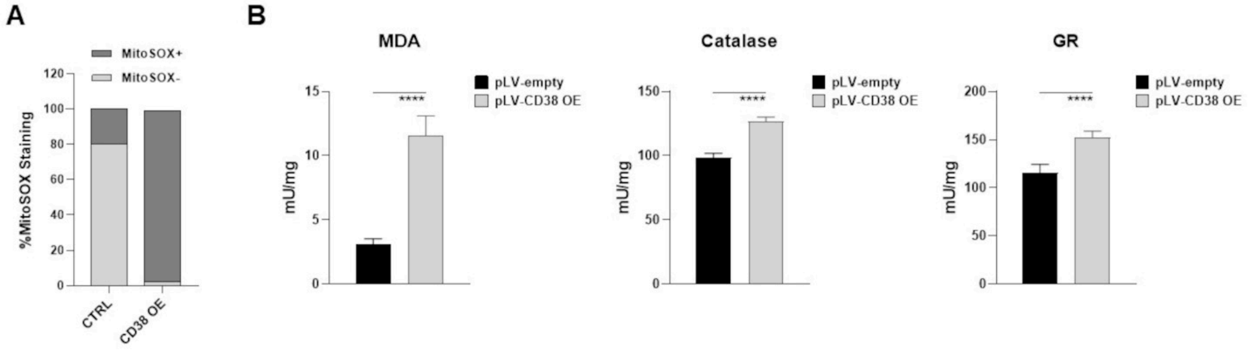

Figure S12. **A)** Mitochondrial superoxide anions were detected by flow cytometry using MitoSOX, in LP1 cells lentivirally transduced with pLVempty vector or pLV CD38 OE. **B)** Oxidative stress marker (MDA) and activities of antioxidant enzymes (Catalase and Glutathione reductase-GR) were measured in LP1 cells lentivirally transduced with pLVempty vector or pLV CD38 OE. Data are ± SD, n=3. (**** p ≤ 0.0001; unpaired t test).

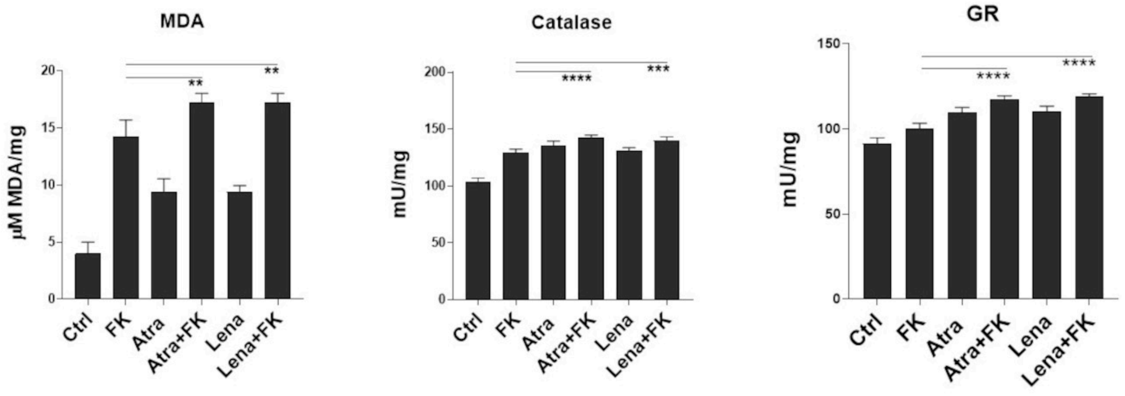

Figure S13. Oxidative stress marker (MDA) and activities of antioxidant enzymes (Catalase, Glutathione reductase-GR) were measured in CD38 inducers (ATRA or Len)-treated cells. Data are presented as mean ± S.D (n=3). (**p≤0.01, *** p= 0.001, **** p ≤ 0.0001; unpaired t test)
