# Supplementary material for: CD38-Induced Metabolic Dysfunction Primes Multiple Myeloma Cells for NAD+-Lowering Agents
Source: Antioxidants (Basel). 2023 Feb 15;12(2):494. doi: 10.3390/antiox12020494 (PMC9952390; doi:10.3390/antiox12020494)
Supplement: Supplementary file 1 [file antioxidants-12-00494-s001.zip › SUPPLEMENTARY METHODS.pdf]

SUPPLEMENTARY METHODS

Viability, apoptosis, and mitosox assay.

The effect of single agents or their combination on MM cell growth was assessed by using CellTiter 96 AQueous One Solution Cell Proliferation Assay system (Promega, Madison, WI, USA), as previously reported.[1] Apoptosis was evaluated by Annexin V/ propidium iodide (PI) and PI staining, respectively, followed by flow cytometric analysis, as previously described.[2] Mitochondiral oxidative stress level was assessed by cytofluorimetric analysis or confocal microscopy after staining with Mitosox reagent (Thermo Fischer Scientific, Waltham, MA, USA) as described in [2].

Immunophenotype for CD38 evaluation

The assessment of the percentage of CD38-expressing CD138-positive, light-chain–restricted multiple myeloma cells is routinely performed for clinical purposes on each BM aspirate from patients with multiple myeloma in the Flow Cytometry Laboratory at the Pathology Unit of IRCCS AOU San Martino-IST, Genoa, Italy. Mean fluorescent intensity for CD38 was evaluated on the CD138-positive light-chain restricted population. Immunophenotipic report assigns a discrete value to each cluster of differentiation (CD): bright expressors are indicated as “++”, lower expressors as “+” and a dim level of expression was categorized among the group of non expressors as “-“. In the graphs we arbitrary assigned the value of 50 to "++" expressors and a value of 10 to "+ ".

Patients with less than 1% of CD138-positive light-chain restricted cells were excluded from our analysis.

Evaluation of intracellular ATP and AMP content

Quantification of ATP and AMP was based on the enzyme coupling method.[3] Twenty micrograms of total proteins was used. Briefly, ATP was assayed spectrophotometrically at 340 nm, following NADP reduction, at 25 °C. The reaction mixture contained the following: 1 mM NADP, 10 mM $MgCl_2$, 5 mM glucose, and 100 mM Tris-HCl, pH 7.4, in 1 ml final volume. Samples were analyzed

before and after the addition of 4 μg purified hexokinase and glucose-6-phosphate dehydrogenase (Sigma-Aldrich, Italy). AMP was assayed spectrophotometrically at 340 nm, following NADH oxidation. The reaction mixture contained the following: 75 mM KCl, 5 mM $MgCl_2$, 0.2 mM ATP, 0.5 mM phosphoenolpyruvate, 0.2 mM NADH, 10 IU adenylate kinase (Sigma-Aldrich, Italy), 25 IU pyruvate kinase plus 15 IU lactate dehydrogenase (Sigma-Aldrich, Italy), and 100 mM Tris-HCl pH 8.0.

Oxygen consumption rate evaluation

Oxygen consumption was measured with an amperometric oxygen electrode (Unisense) in a closed chamber, magnetically stirred, at 37°C. For each assay, 2×10^5 cells were used. After cell permeabilization with 0.03 mg/ml digitonin for 10 min, samples were suspended in 137 mM NaCl, 5 mM KH_2PO_4, 5 mM KCl, 0.5 mM EDTA, 3 mM $MgCl_2$, and 25 mM Tris-HCl, pH 7.4. To activate the pathway composed of complexes I, III, and IV, 5 mM pyruvate and 2.5 mM malate were added. To activate the pathway composed of complexes II, III, and IV, 20 mM succinate was used[4].

Evaluation of F_1F_o-ATP synthase activity

F_1F_o-ATP synthase (ATP synthase) activity was detected by measuring the ATP production by the highly sensitive luciferin/luciferase method. The assays were conducted at 37°C, for 2 min, and data were collected every 30 s. Cells (1×10^5) were added to the incubation medium (0.1 ml final volume), which contained 50 mM KCl, 1 mM EGTA, 2 mM EDTA, 5 mM KH_2PO_4, 2 mM $MgCl_2$, 0.6 mM ouabain, 1 mM P1,P5 -Di(adenosine-5′) pentaphosphate, 0.040 mg/ml ampicillin, 0.2 mM adenosine diphosphate (ADP), 10 mM Tris-HCl pH 7.4, and the respiratory substrates (5 mM pyruvate + 2.5 mM malate or 20 mM succinate). Cells were equilibrated for 10 min at 37°C, and then ATP synthesis was induced by the addition of 0.2 mM ADP. ATP synthesis was measured using the luciferin/luciferase ATP bioluminescence assay kit CLSII (11699695001, Roche) and a Luminometer (GloMax® 20/20 Luminometer, Promega). ATP standard solutions in the concentration range 10^{-10}–10^{-7} M were used for calibration[4].

Respiratory complex activity assay

The activity of the redox complexes I, III, and IV was measured, in a double beam spectrophotometer (UNICAM UV2, Analytical S.n.c.), at 25°C. For each assay, 50 μg total proteins were employed, and the reaction was followed for 5 min, collecting data every 1 min[5]. The enzymatic activity was expressed as mIU/mg total protein (nanomoles/min/mg protein). Complex I (NADH-ubiquinone oxidoreductase) was assayed following the reduction of ferrocyanide, in the presence of NADH, at 420 nm; the reaction mixture contained 30 mM NADH, 40 mM potassium ferrocyanide, and 40 μM antimycin A in 10 mM phosphate buffer pH 7.2. The ferrocyanide extinction coefficient was considered $1 \times 10^{-3}\ M^{-1}\ cm^{-1}$, at 420 nm. Complex III (cytochrome c reductase) activity was measured at 550 nm following the reduction of oxidized cytochrome c. The reaction mixture contained 10 mM phosphate buffer pH 7.2, 0.03% oxidized cytochrome c, and 0.5 mM KCN. Complex IV (cytochrome c oxidase) was assayed following the oxidation of ascorbate-reduced cytochrome c at 550 nm, in a solution containing 10 mM phosphate buffer pH 7.2, 0.03% reduced cytochrome c, and 40 μM antimycin A. In both assays, the cytochrome c extinction coefficient was considered $19.1 \times 10^{-3}\ M^{-1}\ cm^{-1}$, at 550 nm.

Electron Microscopy and quantification of mitochondria morphology parameters

Measurements were generated from transmission electron microscopy images with a magnification of ×20,000. Image J software (version 1.42q, National Institutes of Health, Bethesda, MD) was used to calculate all the mitochondrial size and shape parameters by drawing around of each individual mitochondrion analyzed[6].

In silico dataset analysis

FK866 activity signature was derived from the analysis of GSE96636 (Geo Dataset, https://www.ncbi.nlm.nih.gov/gds); FK866 treated were compared to untreated samples using Limma R package and up-regulated and downregulated genes, selected by p value <0.05 and log2fold change >2 or <-2 cut offs, were used to create activity signatures with the GSVA R package (**Table S1**). Expression of these signatures were evaluated in the MMRF CoMMpass dataset (https://research.themmrf.org). RNA sequencing data of clonal plasma cells from MM patients were

extracted from the Interim Analysis 15 (MMRF_CoMMpass_IA15). Transcript per Million (TPM) reads values, obtained by Salmon gene expression quantification (MMRF_CoMMpass_IA15a_E74GTF_Salmon_Gene_TPM) were used in the GSVA algorithm to transform the gene expression measurements into enrichment scores for the FK866 activity signatures; enrichment scores were then displayed in a heatmap with column clustering.

Samples displaying a signature expression according to FK866 treatment were selected and further divided in quartiles for their expression of CD38. Top and bottom quartiles samples, having highest and lowest CD38 expression, respectively were used to build survival curves. Briefly, Kaplan-Meier method (survival R package) was applied on overall (OS) and progression free (PFS) survival data, long-rank test p-value was calculated between the two groups discriminated by the CD38 gene expression. The same approach was used to stratify CoMMpass dataset samples for their enrichment in oxidative stress response genes expression, by the use of two Gene Ontology biological processes terms: "GOBP_NEGATIVE_REGULATION_OF_RESPONSE_TO_OXIDATIVE_STRESS" and "GOBP_POSITIVE_REGULATION_OF_RESPONSE_TO_OXIDATIVE_STRESS" (GO:1902883, GO:1902884). This analysis identified two groups of samples with negative (LOW) and positive (HIGH) enrichment of response to oxidative stress, further used to build survival curves as specified above. The expression of CD38 (log2 transcript per million reads) among these two groups was represented in scatter plot with bar, created and analyzed with Prism 8 (GraphPad Software).

BIBLIOGRAPHY

1. Soncini D, Orecchioni S, Ruberti S, et al. The new small tyrosine kinase inhibitor ARQ531 targets acute myeloid leukemia cells by disrupting multiple tumor-addicted programs. *Haematologica*. 2020;105(10):2420–2431.

2. Soncini D, Minetto P, Martinuzzi C, et al. Amino acid depletion triggered by L-asparaginase sensitizes MM cells to carfilzomib by inducing mitochondria ROS-mediated cell death. *Blood*

Adv. 2020;4(18):4312–4326.

3. Marini C, Ravera S, Buschiazzo A, et al. Discovery of a novel glucose metabolism in cancer: The role of endoplasmic reticulum beyond glycolysis and pentose phosphate shunt. *Sci. Rep.* 2016;6:.

4. Ravera S, Aluigi MG, Calzia D, et al. Evidence for ectopic aerobic ATP production on C6 glioma cell plasma membrane. *Cell. Mol. Neurobiol.* 2011;31(2):313–321.

5. Bartolucci M, Ravera S, Garbarino G, et al. Functional Expression of Electron Transport Chain and FoF1-ATP Synthase in Optic Nerve Myelin Sheath. *Neurochem. Res.* 2015;40(11):2230–41.

6. Picard M, White K, Turnbull DM. Mitochondrial morphology, topology, and membrane interactions in skeletal muscle: a quantitative three-dimensional electron microscopy study. *J. Appl. Physiol.* 2013;114(2):161–171.
